# Ruthenium Anchored Laser‐Induced Graphene as Binder‐Free and Free‐Standing Electrode for Selective Electrosynthesis of Ammonia from Nitrate

**DOI:** 10.1002/advs.202406843

**Published:** 2024-08-13

**Authors:** Zekun Geng, Zhiliang Feng, Haoran Kong, Jiaqi Su, Kaiyan Zhang, Jiaxin Li, Xinzhi Sun, Xiaojuan Liu, Lei Ge, Panpan Gai, Feng Li

**Affiliations:** ^1^ College of Chemistry and Pharmaceutical Sciences Qingdao Agricultural University Qingdao 266109 China; ^2^ Key Laboratory of Advanced Energy Materials Chemistry (Ministry of Education) Nankai University Tianjin 300071 China

**Keywords:** ammonia, electrocatalysis, laser‐induced graphene, nitrate reduction reaction, ruthenium

## Abstract

Developing effective electrocatalysts for the nitrate reduction reaction (NO_3_RR) is a promising alternative to conventional industrial ammonia (NH_3_) synthesis. Herein, starting from a flexible laser‐induced graphene (LIG) film with hierarchical and interconnected macroporous architecture, a binder‐free and free‐standing Ru‐modified LIG electrode (Ru‐LIG) is fabricated for electrocatalytic NO_3_RR via a facile electrodeposition method. The relationship between the laser‐scribing parameters and the NO_3_RR performance of Ru‐LIG electrodes is studied in‐depth. At −0.59 V_RHE_, the Ru‐LIG electrode exhibited the optimal and stable NO_3_RR performance (NH_3_ yield rate of 655.9 µg cm^−2^ h^−1^ with NH_3_ Faradaic efficiency of up to 93.7%) under a laser defocus setting of +2 mm and an applied laser power of 4.8 W, outperforming most of the reported NO_3_RR electrodes operated under similar conditions. The optimized laser‐scribing parameters promoted the surface properties of LIG with increased graphitization degree and decreased charge‐transfer resistance, leading to synergistically improved Ru electrodeposition with more exposed NO_3_RR active sites. This work not only provides a new insight to enhance the electrocatalytic NO_3_RR performance of LIG‐based electrodes via the coordination with metal electrocatalysts as well as identification of the critical laser‐scribing parameters but also will inspire the rational design of future advanced laser‐induced electrocatalysts for NO_3_RR.

## Graphical abstract

A binder‐free and free‐standing Ru‐anchored laser‐induced graphene (Ru‐LIG) electrode is rationally designed and developed into a high‐performance nitrate reduction electrode, in which the laser‐defocused distance and the laser power are identified as the critical laser‐scribing parameters to tailor the interface properties of the Ru‐LIG electrode and thus improve its activity and selectivity of ammonia electrosynthesis.

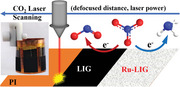

## 1 Introduction

Ammonia (NH_3_), a high‐value critical chemical, is widely used in reactive nitrogen‐based fertilizers for agriculture and is also deemed as a hydrogen‐rich but carbon‐free green feedstock in industry.^[^ ^1^, ^2^, ^3^ ^]^ Renewable electricity‐powered electrocatalytic nitrate reduction reaction (NO_3_RR) using water as a proton source under mild operating conditions represents an appealing approach for green NH_3_ electrosynthesis and has recently been recognized as a decentralized and sustainable alternative strategy to replace the energy‐intensive Haber−Bosch process, offering a great opportunity to balance the nitrogen cycle and to achieve carbon neutrality.^[^ ^4^, ^5^, ^6^, ^7^, ^8^, ^9^ ^]^ Compared with the electrocatalytic N_2_ reduction reaction (NRR),^[^ ^10^, ^11^ ^]^ the use of nitrate as an abundant nitrogen source has the features of much higher water solubility with lower dissociation energy of N ═ O bond, so the reduction of nitrate to NH_3_ at the liquid‐solid interface is thermodynamically more feasible for NH_3_ electrosynthesis than NRR at the solid‐gas‐liquid interface, leading to a high yield rate and Faradaic efficiency (FE) of NH_3_.^[^ ^12^, ^13^, ^14^, ^15^, ^16^, ^17^ ^]^ Moreover, nitrate has become one of the most widespread nitrogen‐containing contaminants in aquatic ecosystems due to excessive fertilization and industrial effluents, which can cause environmental pollution and are highly detrimental to human health. Thus, electrochemical NO_3_RR is not only an energy‐saving strategy for the valorization of nitrate into value‐added NH_3_ as an energy carrier and fertilizer, but also emerges as an economically feasible approach for the effective removal of widespread waste nitrate pollutants at the same time, thus achieving the “Waste‐to‐Wealth” process.^[^ ^18^, ^19^, ^20^, ^21^, ^22^, ^23^, ^24^ ^]^

Recently, laser‐induced graphene (LIG)^[^ ^25^, ^26^ ^]^ has emerged as a useful class of metal‐free electrocatalysts possessing structural features of three‐dimensional (3D) macroporous network architecture endowed with large surface area and abundant defect amorphous structures as active sites, which have attracted increasing attention for the electrocatalysis of various reactions, including glycerol oxidation,^[^ ^27^, ^28^ ^]^ nitrobenzene/CO_2_/oxygen reduction,^[^ ^29^, ^30^, ^31^, ^32^ ^]^ pollutant degradation,^[^ ^33^ ^]^ and water splitting,^[^ ^34^, ^35^, ^36^, ^37^ ^]^ because of its good stability (strong corrosion resistance and wide operating potential range) and easy fabrication. For example, LIG has exhibited excellent electrocatalytic performance in nitrate adsorption and conversion of nitrate to NH_3_ with high FE up to 96.4% and NH_3_ yield rate of 2859 µg cm^−2^ h^−1^ at a relatively high potential of −0.93 V versus reversible hydrogen electrode (RHE).^[^ ^38^, ^39^ ^]^ As is well‐known, NO_3_RR has a complex multistep reduction pathway involving the transfer of eight electrons and nine protons (NO_3_^−^ + 9H^+^ + 8e^−^ → NH_3_ + 3H_2_O). The single LIG electrocatalyst is incapable of simultaneously accelerating the kinetic rate of each intermediate step in the entire NO_3_RR process, and thus it is difficult to achieve competitive reaction kinetics compared to metal electrocatalysts at the same reaction potentials. Fortunately, the electrocatalytic performance of the LIG mono‐electrocatalyst can be improved by introducing other catalytic sites, such as metallic electrocatalysts.^[^ ^34^, ^40^, ^41^, ^42^, ^43^ ^]^ In addition, dispersing metal electrocatalysts into conductive LIG to form isolated electrocatalytic sites exhibits more advantages, such as enhanced activity with higher atomic utilization and nitrate enrichment effect.^[^ ^44^ ^]^ There may also be synergistic effects between metal and LIG^[^ ^40^ ^]^ resulting in more efficient NO_3_RR electrocatalysis. Thus, metal‐LIG is expected to be an attractive NO_3_RR electrocatalyst, which has not yet been reported. Moreover, although the reported ammonia partial current density of LIG can be enhanced by applying high voltage,^[^ ^38^, ^39^ ^]^ LIG has to be scraped from the polyimide (PI) film and then ultrasonically dispersed in Nafion solution for electrode fabrication on other conductive substrates using the drop‐casting method, which is not only adverse to maintain the intrinsic 3D macroporous structure of LIG, but also difficult to fabricate electrodes on a large scale. At the same time, the relationship between the surface properties of LIG and NO_3_RR performance is still not clear. Therefore, the development of NO_3_RR on a binder‐free and free‐standing laser patterned LIG electrode is not only of great significance to study the underlying surface structure mechanism of LIG‐based NO_3_RR electrode but also can further improve its electrocatalytic performance for selective NH_3_ electrosynthesis through rational electrode design and metal electrocatalyst modification.

Here, Ru‐anchored LIG (Ru‐LIG) was demonstrated, for the first time, as an electrocatalyst for NH_3_ electrosynthesis from nitrate electroreduction in a neutral medium. Ru is selected to couple with LIG due to its low nitrate activation barrier.^[^ ^20^, ^45^ ^]^ The Ru‐LIG electrocatalyst is fabricated on the pre‐formed LIG electrode by a facile electrodeposition method, which not only avoids the use of any additional reducing reagents in the whole fabrication process but also enables the uniform and in situ growth of Ru electrocatalysts on the LIG surface for direct contact and rapid transport of reaction intermediates. Importantly, it has been clearly demonstrated that the surface nanostructure, chemical composition, and conductivity of LIG could be tailored by judiciously tuning, for example, the focus distance and the laser power.^[^ ^46^, ^47^, ^48^ ^]^ Therefore, in this work, laser patterning parameters for LIG fabrication on PI films were investigated to understand in particular how the surface properties of LIG affect the electrodeposition process of Ru reactive species as well as the electrocatalytic NO_3_RR performance of Ru‐LIG, minimizing the side NO_3_RR pathways as well as the competing hydrogen evolving reaction (HER). The intrinsic NO_3_RR activities and synergic effects of Ru and LIG enable Ru‐LIG on PI film to act as a superb NO_3_RR electrode for NH_3_ electrosynthesis. In 0.5 M K_2_SO_4_ solution containing 100 mM KNO_3_, such a Ru‐LIG electrode attains a high FE of 93.7% at −0.59 V_RHE_ and a large NH_3_ yield rate of 655.9 µg cm^−2^ h^−1^ at pH 7, not only exhibiting enhanced NO_3_RR activity compared with pristine LIG electrode or Ru nanoparticles (NPs) electrodeposited on a mechanically crushed LIG electrode but also surpassing most previous reports. Furthermore, the Ru‐LIG electrode displays remarkable stability over 10 h successive cycling electrolysis, with nearly unchanged NH_3_ yield rates and FEs.

## 2 Results and Discussion

**Figure 1:**
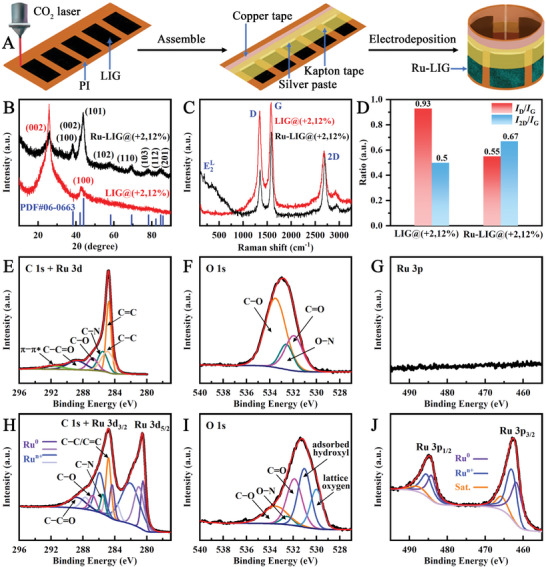
A) Schematic illustration for the fabrication of Ru‐LIG electrode. B) XRD patterns, C) Raman spectra, D) summary of *I*_D_/*I*_G_ and *I*_2D_/*I*_G_ ratios, High‐resolution E, H) C 1s + Ru 3d, F, I) O 1 s, and G, J) Ru 3p XPS of (E‐G) LIG@(+2,12%) electrode and H–J) Ru‐LIG@(+2,12%) electrode.

As illustrated in **Figure** **1**A, all the self‐supported and binder‐free LIG‐based electrodes in this work were fabricated in situ under ambient conditions using a combined strategy based on direct laser patterning and electrodeposition. Typically, a black conductive LIG layer with a size of 1 × 2 cm^2^ was first patterned on a flexible PI film by laser‐scanning at a defocused distance of +2 mm and a laser power of 12% unless otherwise specified, during which the PI film absorbs the laser energy and thus is locally and instantaneously heated to an ultra‐high temperature (>2500 °C), enabling rapid carbonization of the PI surface to LIG at the laser spot. Subsequently, the laser‐patterned LIG layer acts as the electrode substrate for the growth of Ru electrocatalysts through the electrodeposition process. For comparison, the Ru electrocatalysts were also electrodeposited on other LIG electrode substrates prepared at different laser‐patterning parameters, such as different defocused distances (x = 0 and +2 mm) and different laser powers (p = 8%, 10%, 12%, 14%), under the same conditions, denoted as Ru‐LIG@(x,p), to investigate the impact of the LIG electrodes. The X‐ray diffraction (XRD) pattern of the LIG@(+2,12%) electrode (Figure 1B) shows two typical diffraction peaks located at ≈25.5° and 43°, which can be indexed to the (002) and (100) crystal planes of LIG.^[^ ^50^ ^]^ After in situ electrodeposition, eight new diffraction peaks corresponding to the (100), (002), (101), (102), (110), (103), (112), and (201) planes of metallic Ru^0^ (JCPDS card No. 06–0663) were observed (Figure 1B) at 38.39°, 42.15°, 44.00°, 58.32°, 69.40°, 78.39°, 84.70°, and 85.96° without miscellaneous peaks, respectively, suggesting the successful formation of metallic Ru^0^ electrocatalyst on the LIG surface with high purity.

The Raman spectra are then used to evaluate the LIG structural quality of the electrode before and after electrodeposition (Figure 1C). Both the LIG@(+2,12%) and the Ru‐LIG@(+2,12%) electrode exhibit three distinctive Raman bands located at ≈1350, ≈1580, and ≈2690 cm^−1^, corresponding to the D band (representing defective or disordered graphitic lattice structures), G band (indicating the sp^2^ carbon lattice stretching vibration), and 2D band (distinguishing graphene carbon from graphitized and amorphous carbon structures), respectively. The intensity ratio of D to G peaks (*I*_D_/*I*_G_) is usually used to determine the defect degree of graphene material. The calculated *I*_D_/*I*_G_ ratio of the LIG@(+2,12%) electrode was ca. 0.93, indicating the formation of numerous defects in the LIG@(+2,12%) electrode (Figure 1D). The apparent decrease in the *I*_D_/*I*_G_ ratio of the Ru‐LIG@(+2,12%) electrode (0.55) most likely resulted from the reduction of oxygen‐containing groups during the electrodeposition of Ru electrocatalyst, resulting in the improved graphitization of the Ru‐LIG@(+2,12%) electrode. The intensity ratio of 2D and G bands (*I*_2D_/*I*_G_) indicates the number of graphene layers in the multilayered LIG sheets. The *I*_2D_/*I*_G_ ratio of the Ru‐LIG@(+2,12%) electrode was found to be ≈0.67, slightly higher than that of the bare LIG@(+2,12%) electrode (≈0.5), indicating that the multilayer graphene structures of LIG were well maintained without obvious change in the number of graphene layers upon electrodeposition of Ru electrocatalyst. Moreover, the Raman spectrum of the Ru‐LIG@(+2,12%) electrode also shows the characteristic E^L^_2_ vibration peaks of Ru nanoparticles at ≈191 cm^−1^.^[^ ^51^ ^]^ High‐resolution X‐ray photoelectron spectroscopy (XPS) was performed to obtain the detailed chemical composition and valence states of the Ru‐LIG@(+2,12%) electrode. In the high‐resolution C 1s + Ru 3d XPS spectrum for the LIG@(+2,12%) electrode (Figure 1E), the deconvoluted peaks at ≈288.8, 286.8, and 285.5 eV are recognized as C−C ═ O, C−O, and C−N bonds, respectively,^[^ ^52^ ^]^ which agrees well with the deconvolution of the corresponding O 1s (Figure 1F) and N 1s (Figure S1, Supporting Information) core‐level spectra. The peaks centered at the binding energies of 284.8 eV and 285.0 eV are attributed to C−C bonds with sp^2^ and sp^3^ hybridization, respectively. A broad peak is also observed at 291.8 eV (Figure 1E), which is associated with the π−π* shake‐up feature typical of carbon structures.^[^ ^52^ ^]^ As for the high‐resolution C 1s + Ru 3d XPS of the Ru‐LIG@(+2,12%) electrode (Figure 1H), the two sets of doublets centered at ≈280.4/284.3 eV and 282.1/286.0 eV could be assigned to Ru^0^ 3d and Ru^n+^ 3d, respectively,^[^ ^20^, ^53^ ^]^ along with the corresponding satellite doublets at 281.0/285.1 eV and 283.7/287.7 eV. The oxidized state of Ru may be caused by prolonged exposure of the electrocatalyst to air^[^ ^54^ ^]^ or may result from the interaction between Ru and oxygenated functional groups as the anchoring sites for Ru on the surface of the LIG substrate,^[^ ^55^, ^56^ ^]^ which could be further confirmed by the two new peaks arising from the deficient oxygen with hydroxyl (HO) groups adsorbed to the metal Ru^0^ (531.1 eV) and lattice oxygen (529.9 eV) across the O 1s region (Figure 1I) after the electrodeposition of Ru element.^[^ ^57^ ^]^ The Ru 3p signal was further investigated to distinguish the valence state of Ru species (Figure 1J). The Ru 3p orbital XPS profile of the Ru‐LIG@(+2,12%) electrode (Figure 1J) displays two spin‐orbit split doublet peaks at ≈461.8/484.3 eV and 463.2/485.7 eV, respectively, confirming the presence of metallic Ru^0^ and oxidation states (Ru^n+^),^[^ ^53^ ^]^ consistent with the Ru 3d XPS results. These results suggest the successful electrodeposition of Ru nanoparticles embedded in the LIG matrix.

**Figure 2:**
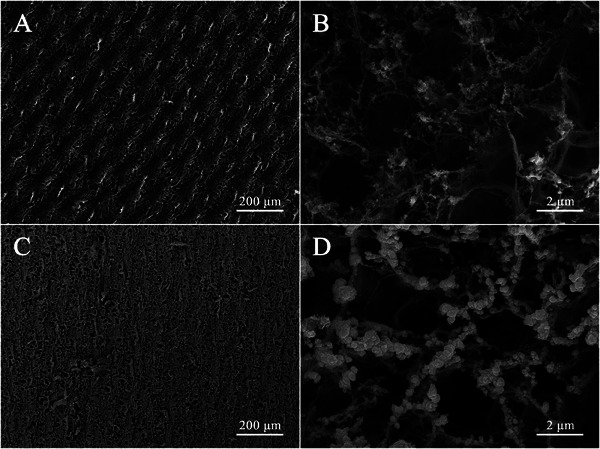
Surface morphology of A, B) LIG@(+2,12%) and C, D) Ru‐LIG@(+2,12%) at different magnifications.

The surface morphologies of the bare LIG@(+2,12%) electrode and the Ru‐LIG@(+2,12%) electrode were further analyzed by scanning electron microscope (SEM). As displayed in **Figure** **2**A, the low‐resolution SEM image of the bare LIG@(+2,12%) electrode shows a 3D wave‐like macroporous structure with numerous uniformly distributed micro‐pores due to the rapid heating and outgassing during the laser‐scribing process. The magnified SEM image of the LIG@(+2,12%) electrode in Figure 2B exhibits many hierarchical pores embedded in the multilayered and interconnected graphene flakes to form a 3D microporous foam‐like framework. The SEM image of the Ru‐LIG@(+2,12%) electrode at low magnification (Figure 2C) demonstrates that the entire surface of the Ru‐LIG@(+2,12%) electrode is uniformly covered with a thin layer of Ru nanoparticles. The larger magnification SEM image of the Ru‐LIG@(+2,12%) electrode (Figure 2D) shows that the exposed surfaces of the multilayered graphene flakes are fully decorated by bushy Ru nanoparticles and thus become relatively rough, with some larger Ru nanoparticles (50–500 nm) grown on the edge‐portion of graphene flakes. The uniform distribution of Ru nanoparticles in the Ru‐LIG@(+2,12%) electrode is further confirmed by elemental mapping (Figure S2, Supporting Information). After electrodeposition, the Ru‐LIG@(+2,12%) electrode well maintains the 3D hierarchically macroporous structural features of the bare LIG@(+2,12%) electrode.

**Figure 3:**
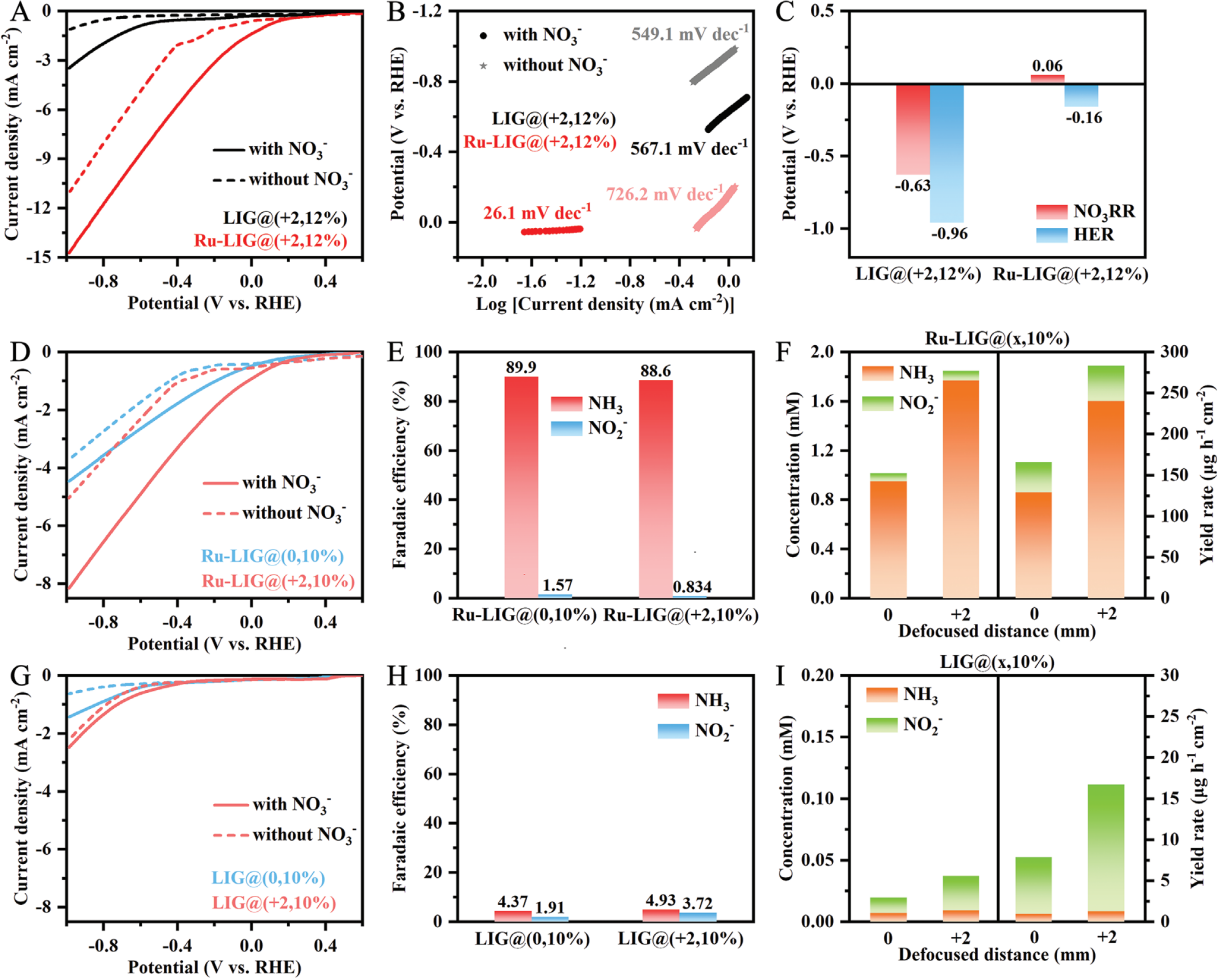
A) LSV curves (without *iR* corrected) and B) Tafel curves over LIG@(+2,12%) and Ru‐LIG@(+2,12%) electrode at a scan rate of 2.0 mV s^−1^. C) Required potentials to drive 1.0 mA cm^−2^ cathodic current of NO_3_RR and HER over LIG@(+2,12%) and Ru‐LIG@(+2,12%) electrode. D, G) LSV curves, E, H) product FEs, F, I) product distribution, and corresponding yield rate of LIG@(x,10%) and Ru‐LIG@(x,10%) electrodes at an applied potential of −0.39 V_RHE_. All experiments were carried out in Ar‐saturated 0.5 M K_2_SO_4_ electrolyte with/without 100 mM KNO_3_.

The NO_3_RR performance of the laser‐induced electrodes was then assessed under ambient conditions. **Figure** **3**A shows the linear sweep voltammetry (LSV) curves of the bare LIG@(+2,12%) and the Ru‐LIG@(+2,12%) electrodes in K_2_SO_4_ solution with and without 100 mM NO_3_^−^. In the absence of NO_3_^−^, the Ru‐LIG@(+2,12%) electrode exhibits a higher HER activity compared to the LIG@(+2,12%) electrode. The reduction peaks of the Ru‐LIG@(+2,12%) electrode at ≈−0.35 V_RHE_ can be partially attributed to the reduction of surface Ru oxide. It has been reported that moderate HER activity with appropriate hydrogen supply capability assists in providing protons for facilitating the subsequent electrocatalytic hydrogenation reaction of NO_2_^−^ intermediates, benefiting NH_3_ electrosynthesis.^[^ ^58^ ^]^ After the addition of NO_3_^−^, the bare LIG@(+2,12%) electrode shows only a slightly enhanced electrocatalytic current density, indicating its weak NO_3_RR activity. In contrast, a significant enhancement of the current density is observed over the Ru‐LIG@(+2,12%) electrode in the presence of NO_3_^−^, confirming that Ru as an active electrocatalyst enables higher electrocatalytic activity toward NO_3_RR. In addition, the Ru‐LIG@(+2,12%) electrode achieves a much smaller Tafel slope (26.1 mV dec^−1^) than that of the LIG@(+2,12%) electrode (567.1 mV dec^−1^) (Figure 3B) in the presence of NO_3_^−^, reflecting kinetically faster NO_3_RR process on Ru‐LIG. Moreover, the onset potential, which is defined as the applied potential to reach a cathodic current of 1.0 mA cm^−2^, of the Ru‐LIG@(+2,12%) electrode in the presence of NO_3_^−^ is 0.06 V_RHE_ (Figure 3C), while the onset potential of the LIG@(+2,12%) electrode is −0.63 V_RHE_. Therefore, NO_3_RR is more likely to occur on the Ru‐LIG@(+2,12%) electrode than the competitive HER, and the Ru‐LIG@(+2,12%) electrode exhibits higher NO_3_RR activity with a lower overpotential compared with the LIG@(+2,12%) electrode.

To understand the key factors of the LIG substrate that influence the NO_3_RR activity of the Ru‐LIG electrode in‐depth, the laser‐patterning parameters of the LIG substrate need to be investigated based on maximizing the resultant NO_3_RR performance. As a critical parameter, the influence of laser‐defocused distance on NO_3_RR products is first exploited by changing the vertical distance between the laser focal point and the PI surface (including 0 and +2 mm) and fixing other parameters (i.e., 10% of full scan rate, 10% of full laser power, 1200 DPI (dots per inch)). LSV curves were recorded before the chronoamperometric tests to verify the NO_3_RR activity of different electrodes. As displayed in Figure 3D,G, all electrodes fabricated at different defocused distances exhibit enhanced current densities in the presence of NO_3_^−^. Before electrodeposition, the LIG@(0,10%) electrode fabricated at the focused distance shows a lower current density but a higher current change compared to that of the LIG@(+2,10%) electrode (Figure 3G). However, a positive correlation between the current density/change and the defocused distance is clearly observed for all Ru‐LIG(x,10%) electrodes (Figure 3D), in which the Ru‐LIG@(+2,10%) electrode shows higher current density and maximum current change. The concentrations of the NO_3_RR products, including NO_2_^−^ and NH_3_, produced during the chronoamperometric tests at −0.39 V_RHE_ (Figure S3, Supporting Information) under ambient conditions were analyzed using colorimetric methods (Figures S4 and S5, Supporting Information). As expected, although the Ru‐LIG@(0,10%) and the Ru‐LIG@(+2,10%) electrodes exhibit similar NH_3_ FE (Figure 3E), the Ru‐LIG@(+2,10%) electrode has a much higher NH_3_ yield rate of 240.5 µg cm^−2^ h^−1^, which is ≈1.86 times higher than that of the Ru‐LIG@(0,10%) electrode (Figure 3F). In contrast, the generated amount of NH_3_ over the bare LIG@(x,10%) electrodes also increases with the increase of the defocused distance but shows much lower NH_3_ FE (<5%, Figure 3H) and yield rate (<2.5 µg cm^−2^ h^−1^, Figure 3I) than those of the Ru‐LIG@(x,10%) electrodes under the same conditions due to the low NO_3_RR activity of the bare LIG@(x,10%) electrodes. As a significant intermediate during NO_3_RR, the concentration of generated NO_2_^−^ over the bare LIG@(x,10%) and the Ru‐LIG@(x,10%) electrodes both increased with the increasing of the defocused distance (Figure 3F,I). Compared with the bare LIG@(x,10%) electrode, the relative NO_2_^−^ selectivity over the Ru‐LIG@(x,10%) electrodes is much lower (Figure 3F,I), indicating that the generated NO_2_^−^ could be consumed more rapidly on the Ru‐LIG@(x,10%) electrodes. These results suggest the key role of the defocused distance in the NO_3_RR of the Ru‐LIG electrode.

**Figure 4:**
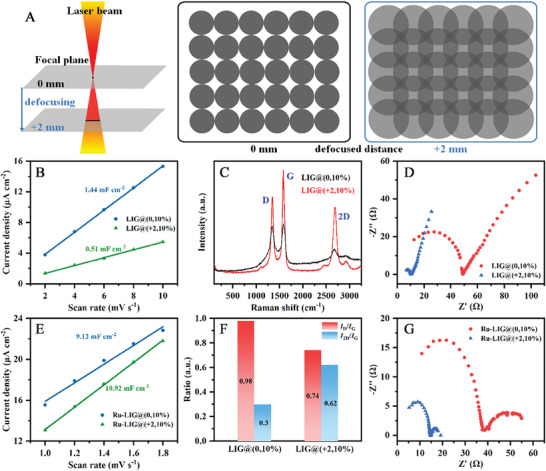
A) Schematic illustration for the laser defocusing. The capacitive currents of B) LIG@(x,10%) electrode and E) Ru‐LIG@(x,10%) electrode measured at ≈0.91 V_RHE_ plotted as a function of scan rate. C) Raman spectra and F) summary of *I*_D_/*I*_G_ and *I*_2D_/*I*_G_ ratios of LIG@(x,10%) electrode. EIS measurement of D) LIG@(x,10%) electrode and G) Ru‐LIG@(x,10%) electrode at an applied potential of −0.39 V_RHE_ in Ar‐saturated 0.5 M K_2_SO_4_ electrolyte with/without 100 mM KNO_3_ over a range of 10^6^ to 10^−1^ Hz.

**Figure 5:**
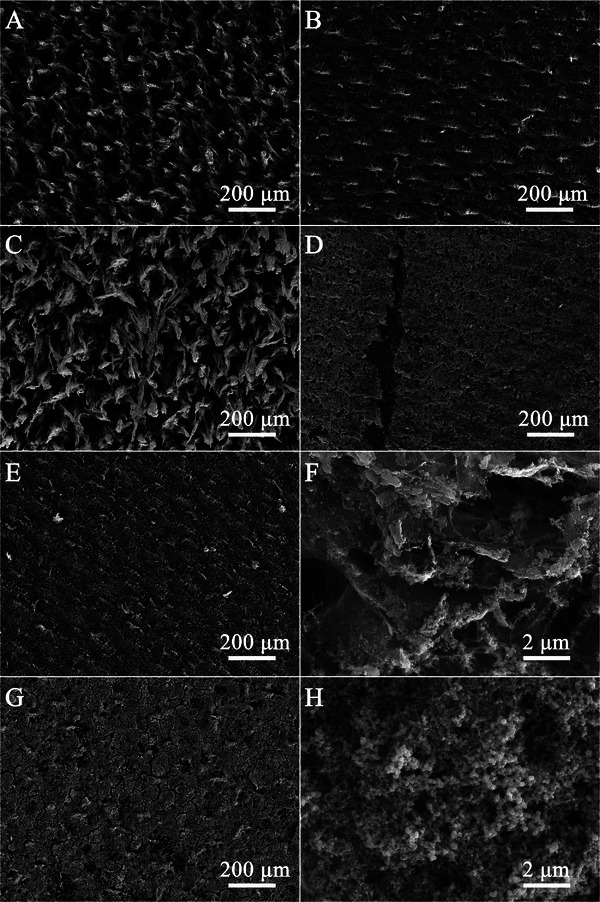
SEM images of A) LIG@(0,10%), B) LIG@(+2,10%), C) Ru‐LIG@(0,10%), and D) Ru‐LIG@(+2,10%) electrodes at low magnification. SEM images of (E,F) cLIG@(+2,12%) and G, H) Ru‐cLIG@(+2,12%) electrodes at different magnification.

As reported previously, altering the defocused distance can cause the laser spot size to vary due to the conical shape of the laser beam.^[^ ^59^, ^60^ ^]^ When the PI substrate moves far away from the focal point, the laser spot size is enlarged (**Figure** **4**A), resulting in not only the occurrence of overlapping scanning but also the decrease of the laser power density irradiated on the PI surface, which further changes the surface properties of LIG, such as the electrochemical active surface area (ECSA) and the charge transfer resistance, and thus affects both the loading mass and the distribution of Ru electrocatalysts. To probe the origin of the high NO_3_RR performance on the Ru‐LIG@(x,10%) electrodes fabricated under different defocused distances, their ECSAs were first estimated by calculating the electrochemical double‐layer capacitances (*C*_dl_, Figure S6, Supporting Information), which are positively correlated with the ECSA. As shown in Figure 4B, the *C*_dl_ of the LIG@(0,10%) electrode (1.44 mF cm^−2^) is ≈3 times higher than that of the LIG@(+2,10%) electrode (0.51 mF cm^−2^). In contrast, the *C*_dl_ of the Ru‐LIG@(+2,10%) electrode (10.92 mF cm^−2^) is slightly larger than that of the Ru‐LIG@(0,10%) electrode (9.12 mF cm^−2^)(Figure 4E), which may promote the exposure of more available active sites as well as the diffusion of reactive species, thus benefiting the NO_3_RR as shown in Figure 3. The above results suggest that the contribution of the LIG substrate to the NO_3_RR performance of the Ru‐LIG electrode is not due to the improvement in the ECSA of the LIG substrate. Moreover, the high *C*_dl_ of the LIG@(0,10%) electrode could be attributed to the higher level of structural defects and thicker stacking, as indicated by the larger *I*_D_/*I*_G_ ratio and the smaller *I*_2D_/*I*_G_ ratio, respectively, in the Raman spectra (Figure 4C,F).^[^ ^39^ ^]^ When the laser is defocused to +2 mm distance, an obvious decrease in the *I*_D_/*I*_G_ ratio with narrowing of both D and G peaks is observed, which may be attributed to the evolution of the surface morphology from forest‐like fibrous microstructures (Figure 5A; Figure S7A, Supporting Information) with highly defected/disordered carbon and stacking faults to foam‐like microporous framework (Figure 5B; Figure S7B, Supporting Information) with high degree of graphitization and LIG formation.^[^ ^26^ ^]^ Meanwhile, the surface morphology transition has a dominant influence on the wettability of the electrode surface. As expected, the LIG@(+2,10%) electrode with a flat and foam‐like microporous morphology promotes the solid‐liquid surface contacting, showing a remarkably decreased contact angle from 95.2° to 0° (Figure S8, Supporting Information), which is in good agreement with previous reports.^[^ ^61^, ^62^ ^]^ After the electrodeposition of Ru, the forest‐like surface morphology of the LIG@(0,10%) electrode is maintained, but small Ru nanoparticles are sparsely grown on the LIG fibers (Figure 5C; Figure S7C, Supporting Information). In the case of the Ru‐LIG@(+2,10%) electrode, a dense and thick layer of Ru nanoparticles was observed covering the entire surface of the Ru‐LIG@(+2,10%) electrode (Figure 5D; Figure S7D, Supporting Information), indicating a large amount of Ru mass loaded on the LIG@(+2,10%) electrode with better hydrophilicity. On the other hand, the fewer structural defects as well as the higher surface wettability potentially result in a faster interface charge‐transfer kinetics. To confirm this, the electrochemical impedance spectroscopy (EIS) of these electrodes fabricated at different defocused distances was measured in 0.5 M K_2_SO_4_ electrolyte containing 100 mM KNO_3_ at −0.39 V_RHE_ to evolute the charge transfer properties of these electrodes. The obtained Nyquist plots of these electrodes in Figure 4D,G presents two semi‐circles, corresponding to the charge transfer resistance in the low‐frequency zone and the passivated film impedance in the high‐frequency region, respectively.^[^ ^39^ ^]^ As displayed in Figure 4D,G, the radius of both semi‐circles over the LIG@(0,10%) and the Ru‐LIG@(0,10%) electrodes fabricated at the focused distance are much larger than that of the electrodes fabricated at a defocused distance of +2 mm under the same conditions, indicating that defocusing leads to the decrease of both the passivated film impedance and the electron transfer resistance, thereby promoting the subsequent electrodeposition of Ru as shown in Figure 5D. The above results reveal that laser defocusing plays a significant role in promoting the charge transfer process across the Ru‐LIG/electrolyte interface as well as the Ru loading of the Ru‐LIG electrode, both of which could provide Ru‐LIG electrode with abundant electrocatalytic NO_3_RR active sites and thus is conducive to high NO_3_RR performance.

**Figure 6:**
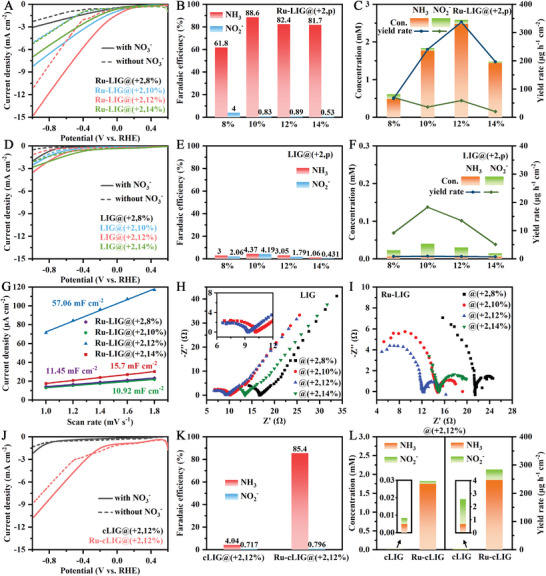
A,D,J) LSV curves, B,E,K) product FEs, C,F,L) product distribution and corresponding yield rate of A,B,C) Ru‐LIG@(+2,p), D,E,F) LIG@(+2,p), J,K,L) cLIG@(+2,12%) and Ru‐cLIG@(+2,12%) electrodes at an applied potential of −0.39 V_RHE_. (G) The capacitive currents of Ru‐LIG@(+2,p) electrodes measured at ≈0.91 V_RHE_ plotted as a function of scan rate. EIS measurement of (H) LIG@(+2,p) electrode and I) Ru‐LIG@(+2,p) electrode at an applied potential of −0.39 V_RHE_ over a range of 10^6^ to 10^−1^ Hz. All experiments were carried out in Ar‐saturated 0.5 M K_2_SO_4_ electrolyte with/without 100 mM KNO_3_. The full laser power is 40 W.

As another key parameter of laser patterning, the influence of laser power on NO_3_RR performance of the Ru‐LIG@(+2,p) electrode is further studied. The LSV curves of the Ru‐LIG@(+2,p) electrodes fabricated at different laser powers ranging from 8% to 14% are shown in **Figure** **6**A. The increase of the current intensity was observed with the increase of the laser power from 8% to 12%, while the further increase of the laser power led to an obvious decline of the current intensity, indicating that the Ru‐LIG@(+2,12%) electrode has the highest NO_3_RR activity. Figure 6B,C show the FEs and the yield rates of the Ru‐LIG@(+2,p) electrodes over the investigated laser power range after chronoamperometric tests at −0.39 V_RHE_ (Figure S9A,B, Supporting Information) under ambient conditions. The similar and significantly high NH_3_ FEs (> 80%) under different laser powers of 10%, 12%, and 14% indicate the high NH_3_ selectivity of the Ru‐LIG@(+2,p) electrodes (Figure 6B). Notably, although the Ru‐LIG@(+2,10%) electrode has a slightly better FE of 88.6%, the highest NH_3_ yield up to 337.4 µg cm^−2^ h^−1^ is observed at 12% laser power (Figure 6C) with an FE of 82.4%. As shown in Figure 6D, the LIG electrodes fabricated at different laser powers show similar current densities and are also capable of producing NH_3_ by electrocatalytic NO_3_RR, but offer much lower FE (<5%, Figure 6E) and NH_3_ yield (<2.0 µg cm^−2^ h^−1^, Figure 6F), reflecting that the promoted NH_3_ production under the laser power of 12% may be also achieved by the improvement of the Ru electrodeposition, which can be reflected by their SEM images. With the laser power increased from 10% to 12%, the surface morphology of the LIG@(+2,12%) electrode shows a hierarchical and interconnected macroporous architecture, which is composed of larger LIG flakes with fibrous edges (Figure 2B) compared with the LIG@(+2,10%) electrode (Figure S7B, Supporting Information), exhibiting much larger pore size. Moreover, compared to the densely covered surface of the Ru‐LIG@(+2,10%) electrode, which is completely filled with numerous Ru nanoparticles (Figure 5D; Figure S7D, Supporting Information), the continuous macroporous network architecture of the LIG@(+2,12%) electrode (Figure 2B) is well‐preserved after Ru electrodeposition (Figure 2D), benefiting the exposure of more NO_3_RR active sites in the Ru‐LIG@(+2,12%) electrode, which is further verified by the remarkably high *C*_dl_ value of the Ru‐LIG@(+2,12%) electrode shown in Figure 6G and Figures S10–S12 (Supporting Information). Considering the slight change in impedance spectroscopy (Figure 6H,I) as well as the negligible change in wettability (Figure S8, Supporting Information) between 10% and 12% laser power, the prominent morphology change should play a significant role in the enhanced NO_3_RR performance of the Ru‐LIG@(+2,12%) electrodes. To verify that the 3D macroporous interface of the LIG@(+2,12%) electrode is indispensable for the electrocatalytic NO_3_RR performance of the Ru‐LIG@(+2,12%) electrode, the NO_3_RR performance of the Ru‐cLIG@(+2,12%) electrode prepared from the mechanically crushed LIG@(+2,12%) (denoted as cLIG@(+2,12%)) was investigated for comparison. The mechanically crushed surface of cLIG@(+2,12%) was obtained by pressing a glass slide with 3.0 kg weight atop the pristine LIG@(+2,12%) surface for 12 h. SEM was then carried out to characterize the surface morphology of cLIG@(+2,12%) (Figure 5E), which shows that cLIG@(+2,12%) has a flatter surface with crushed LIG flakes (Figure 5F). After the electrodeposition of Ru, cLIG with a flattened surface leads to the preferred formation of a dense and thick layer of Ru nanoparticles, which thus completely covered/filled the entire rough interface of cLIG@(+2,12%) (Figure 5G,H), resulting in a flattened surface like that of the Ru‐LIG@(+2,10%) electrode (Figure 5D). The potentiostatic electrocatalysis results of NO_3_RR at −0.39 V_RHE_ show that, although the FE is slightly higher (85.4% in Figure 6K), the current density (ca. 4.0 mA cm^−2^ in Figure S9C, Supporting Information) and NH_3_ yield rate (ca. 247.1 µg cm^−2^ h^−1^ in Figure 6L) of the Ru‐cLIG@(+2,12%) electrode are significantly lower than those of the Ru‐LIG@(+2,12%) electrode (ca. 10.0 mA cm^−2^ in Figure S9B (Supporting Information) and 337.4 µg cm^−2^ h^−1^ in Figure 6C). Moreover, the inferior NO_3_RR performance of the Ru‐cLIG@(+2,12%) electrode is observed more clearly at higher applied potential (Figure S13, Supporting Information), confirming that the hierarchical and interconnected macroporous architecture of the LIG@(+2,12%) electrode is an important factor for the high NO_3_RR property of Ru‐LIG@(+2,12%), which probably provides a good synergistic effect between LIG and the electrodeposited Ru nanoparticles.

**Figure 7:**
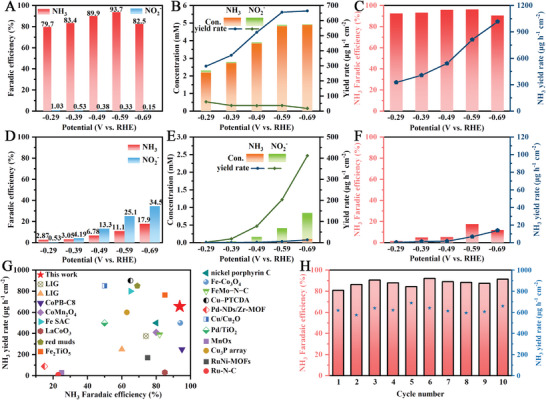
A,D) Product FEs, B,E) product distribution, and corresponding yield rate of (A,B) Ru‐LIG@(+2,12%) and D,E) LIG@(+2, 12%) electrodes at different applied potentials. NH_3_ yield rate and FEs of NO_2_RR over (C) Ru‐LIG@(+2,12%) and (F) LIG@(+2, 12%) electrode at different applied potentials. (G) Comparison of the electrocatalytic NO_3_RR performances of Ru‐LIG@(+2,12%) electrode with other reported electrocatalysts under similar conditions. (H) NH_3_ yield rate and FEs of Ru‐LIG@(+2,12%) electrode for ten consecutive NO_3_RR recycling tests at an applied potential of −0.59 V_RHE_, the cycle time was 1 h. All experiments were carried out in Ar‐saturated 0.5 M K_2_SO_4_ electrolyte with 100 mM KNO_3_ or NaNO_2_.

The LSV results in Figure 3A suggest that the Ru‐LIG@(+2,12%) electrode can effectively reduce NO_3_^−^ over a wide potential range from 0.0 V_RHE_ to −1.0 V_RHE_, where the current density elevated significantly in the electrolyte with NO_3_^−^. Thus, to investigate the effect of the applied potential on the yield rates and FEs toward NH_3_, chronoamperometric tests (Figure S14, Supporting Information) were performed using the Ru‐LIG@(+2,12%) electrode or the LIG@(+2,12%) electrode as the working electrode at a series of operating potentials ranging from −0.29 V_RHE_ to −0.69 V_RHE_. As shown in **Figure** **7**A, the NH_3_ FE over the Ru‐LIG@(+2,12%) electrode initially increases and then decreases as the applied cathodic potential increases, reaching a maximum value of 93.7% at −0.59 V_RHE_. The decrease in NH_3_ FE at higher applied cathodic potential is mainly due to the competitive HER. However, the NH_3_ yield rate over the applied potential range shows an increasing trend over the whole investigated potential range (Figure 7B) and reaches a high value of 664.6 µg cm^−2^ h^−1^ at −0.69 V_RHE_. Furthermore, in the case of the LIG@(+2,12%) electrode, the predominant reduction product is NO_2_^−^ over the entire applied potential range (Figure 7D,E). Notably, both the FE (Figure 7D) and the yield rate (Figure 7E) of NO_2_^−^ increased remarkably with the increase of the cathodic potential, suggesting that the LIG@(+2,12%) electrode preferentially produces NO_2_^−^ rather than NH_3_ at high cathodic potentials. In comparison, the Ru‐LIG@(+2,12%) electrode shows a much better NO_2_RR performance with excellent NH_3_ yield rate (813.2 µg cm^−2^ h^−1^) and FE (96.3%) (Figure 7C) than that of the LIG@(+2,12%) electrode (Figure 7F) during the chronoamperometric tests at different applied potentials (Figure S15, Supporting Information). Hence, the production of NO_2_^−^ in NO_3_RR over the Ru‐LIG@(+2,12%) electrode (Figure 7A,B) is gradually inhibited with the increase of the applied voltage, implying that the generated NO_2_^−^ in NO_3_RR process could be quickly converted to NH_3_ in the 3D macroporous structure of the Ru‐LIG@(+2,12%) electrode due to the fast conversion rate of NO_2_^−^ to NH_3_. Thus, the escape of *NO_2_^−^ from the Ru‐LIG@(+2,12%) electrode into the electrolyte is greatly suppressed in the 3D macroporous structure, leading to a remarkably declined generation of the intermediate product NO_2_^−^ ions. Considering both the NH_3_ yield rates and FE, −0.59 V_RHE_ is selected as the optimum potential for the Ru‐LIG@(+2,12%) electrode with a high NH_3_ yield rate of 655.9 µg cm^−2^ h^−1^, an optimal FE of 93.7% for NH_3_ production (Figure 7).

Taking into account of the applied voltage (ca. −0.59 V_RHE_), pH (ca. 7), and the initial NO_3_^−^ concentration (ca. 100 mM), the Ru‐LIG@(+2,12%) electrode exhibits superior or competitive NO_3_RR performance in comparison to many previously reported values of NO_3_RR electrocatalysts, as displayed in Figure 7G and Table S1 (Supporting Information).^[^ ^8^, ^23^, ^38^, ^39^, ^43^, ^63^, ^64^, ^65^, ^66^, ^67^, ^68^, ^69^, ^70^, ^71^, ^72^, ^73^, ^74^, ^75^, ^76^ ^]^ The electrodeposition of Ru on the 3D macroporous interface of LIG@(+2,12%) will not only facilitate the N‐O bonds cleavage but also be beneficial for the capture of the escaped NO_2_^−^ due to the large number of exposed NO_3_RR active sites as well as the fast mass transfer in the 3D macroporous structure of LIG. The electrocatalytic NO_3_RR performance of the Ru‐LIG@(+2,12%) electrode for higher NO_3_^−^ concentrations was also explored by chronoamperometric tests at the optimal potential (−0.59 V_RHE_) in K_2_SO_4_ solution with 0.1 M to 0.9 M NO_3_^−^ (Figure S16A, Supporting Information). As displayed in Figure S16B (Supporting Information), the NH_3_ yield rate of the Ru‐LIG@(+2,12%) electrode increased remarkably with the increase of NO_3_^–^ concentration, suggesting that the NH_3_ formation can be effectively fascinated by the increased NO_3_^–^ concentration. When the nitrate concentration is up to 0.9 M, the NH_3_ yield rate increases linearly and reaches 978.4 µg cm^−2^ h^−1^. Besides, the maximal NH_3_ FE (97.9%) in the tested nitrate concentration range is observed at 0.3 M nitrate (Figure S16C, Supporting Information) with a NH_3_ yield rate of 732.2 µg cm^−2^ h^−1^, suggesting an extremely high selectivity of NH_3_ electrosynthesis. In the range of 0.3 to 0.9 M, the Ru‐LIG@(+2,12%) electrode shows a slight decrease in FE (from 97.9% to 94.3%) possibly due to that the active sites are occupied by abundant NO_3_^–^, which leads to a limited proton supply, as evident by the obviously increased accumulation of the intermediate product NO_2_^–^ (Figure S16C, Supporting Information). Therefore, the Ru‐LIG@(+2,12%) electrode still maintains appreciable NH_3_ FE and yield rate under higher nitrate concentration, indicating that the Ru‐LIG@(+2,12%) electrode has potential applications under high nitrate concentrations. Finally, the durability of the Ru‐LIG@(+2,12%) electrode was investigated by performing consecutive cycling electrolysis at the optimal potential (−0.59 V_RHE_) (Figure 7H, each cycle for 1.0 h) in K_2_SO_4_ solution with 100 mM NO_3_^−^. Figure S17 (Supporting Information) displays the corresponding chronoamperometric curves. Note that both the yield rates and the FEs of NH_3_ are well preserved without any apparent decay trend after 10 successive recycling tests, indicating the excellent long‐term stability of the Ru‐LIG@(+2,12%) electrode for efficient NO_3_RR.

## 3 Conclusion

In summary, a model and efficient LIG‐based NO_3_RR electrocatalyst was developed by the in situ electrodeposition of Ru on LIG and then used as a binder‐free and free‐standing electrode for highly selective electrocatalytic nitrate reduction to synthesize NH_3_. The self‐supported monolithic Ru‐LIG electrode proposed here is more advantageous than the conventional LIG‐based NO_3_RR electrocatalyst, because it not only simplifies the electrode fabrication but also retains the intrinsic 3D macroporous structure of the LIG film, beneficial for investigating the relationship between the surface properties of LIG and the NO_3_RR performance, which has never been reported before. Therefore, the influence of the laser parameters during the laser‐scribing process on the electrocatalytic NO_3_RR performance of the Ru‐LIG electrode was investigated in this work, and the remarkably enhanced NO_3_RR performance of the Ru‐LIG@(+2,12%) electrode verifies the criticality of laser defocused distance and laser power. Raman, SEM, and EIS characterizations reveal that the surface morphology of LIG undergoes an evolution from forest‐like fibrous microstructures to foam‐like microporous framework with a higher degree of graphitization and LIG formation as well as lower charge transfer resistance when the laser is defocused from 0 to +2 mm distance. Moreover, larger LIG flakes with fibrous edges are observed when the laser power is changed from 10% to 12%, which has been shown to be favorable for maintaining the hierarchical and interconnected macroporous architecture after Ru electrodeposition, thus favoring the dispersion of Ru nanoparticles as well as the exposure of more NO_3_RR active sites. As a result, the Ru‐LIG@(+2,12%) electrode exhibits a highly selective and stable NH_3_ generation with a maximum NH_3_ FE of 93.7% and a yield rate of 655.9 µg cm^−2^ h^−1^ at −0.59 V_RHE_, outperforming most of the reported NO_3_RR electrodes operated under similar conditions, whereas the LIG@(+2,12%) electrode prefers NO_2_^–^ formation under the same conditions. This study not only promotes the NO_3_RR performance of LIG by electrodepositing metal Ru into LIG as effective active sites but also proposes the rational optimization of laser‐scribing parameters to develop Ru‐LIG into a high‐performance NO_3_RR electrode for electrocatalytic NO_3_RR.

## 4 Experimental Section

### Reagents and Materials

Ruthenium(III) chloride (RuCl_3_, 95%, Aladdin), potassium sulfate (K_2_SO_4_, 99%, Aladdin), ammonium chloride (NH_4_Cl, 99.5%, Aladdin), potassium nitrate (KNO_3_, 99%, Aladdin), sodium nitrite (NaNO_2_, 99%, Aladdin), sodium hydroxide (NaOH, 99%, Aladdin), salicylic acid (99%, Aladdin), sodium citrate (99%, Aladdin), sodium nitroferricyanide dihydrate (99%, Aladdin), sodium hypochlorite (99%, Aladdin), N‐(1‐naphthyl) ethylenediamine dihydrochloride, sulfonamide (99%, Aladdin), and phosphoric acid (H_3_PO_4_, 99%, Aladdin) were used without further purification. Kapton polyimide (PI) sheets (500HN, thickness: 125 µm) were purchased from DuPont. Prior to use, the PI sheets were ultrasonically cleaned in acetone, ethanol, and water, respectively, for 30 min each, followed by copious rinsing with ultrapure water, and blown dry under a stream of N_2_ gas. Ultrapure water (resistivity of 18.2 MΩ cm at 25 °C) was provided by a Milli‐Q water purification system (Millipore Corp., Bedford, MA, USA).

### Fabrication of Ru‐LIG Electrode

First, PI film was directly scribed by a 10.6 µm CO_2_ laser (Epilog Laser engraving machine) under ambient conditions at a fixed DPI (1200) and scan rate (10% of the full scan rate) to straightforwardly carbonize its surface into LIG film with a designed pattern (1 × 2 cm^2^ rectangular). For comparison study, LIG electrodes were prepared under different defocused distances or laser powers. Next, conductive colloidal silver ink and copper tape were applied at one end of the rectangular LIG film to improve the conductivity between the electrode and the electrochemical workstation for subsequent electrodeposition or electrochemical testing. A Kapton PI tape was then employed not only to protect the copper tape and silver paint from the electrolyte but also to maintain the effective working area of the square‐shaped LIG electrode at 1.0 cm^2^. Electrodeposition of Ru on the LIG electrode was achieved using a three‐electrode setup by immersing the LIG electrode into an electrolyte bath containing 1.0 M NaCl and 6.0 mM RuCl_3_ with Ag/AgCl (with saturated KCl solution) as the reference electrode and a platinum wire as the counter electrode. The electrodeposition was performed at a constant current of −20 mA for 20 min at room temperature. After electrodeposition, the Ru‐LIG electrode was immersed in ultrapure water for 30 min to remove excess electrolytes and dried under vacuum overnight.

### Characterizations

X‐ray diffraction (XRD) patterns were collected using a Bruker D8 diffractometer (Germany) with Cu Kα as the radiation source over the 2*θ* scan range of 20−70°. X‐ray photoelectron spectroscopy (XPS) measurements were performed on an ESCALAB 250Xi instrument (Thermo Fisher, Waltham, MA, Al Kα radiation). Scanning electron microscope (SEM) images were recorded using a FEI QUANTA Q400 electron microscopy operating at an accelerating voltage of 20 kV. Raman spectra were measured by a DXRxi Raman spectrometer (Thermo Scientific) with an excitation wavelength of 532 nm. Static water contact angles were measured at room temperature by means of a standard contact‐angle measuring instrument (KRUSS DSA25) employing drops of pure water.

### Electrochemical Measurements

All electrochemical measurements for nitrate reduction were carried out in a typical three‐electrode configuration using Ar‐saturated 0.5 M K_2_SO_4_ electrolyte with/without 100 mM KNO_3_ at 25 ± 1 °C and ambient pressure. CHI 760E electrochemical workstation (CH Instruments, Inc.) was employed to record the results of all electrochemical responses without *iR*‐correction. Prior to each nitrate electroreduction test, linear sweep voltammetry (LSV) curves were recorded until the polarization curves reached steady‐state at a rate of 2.0 mV s^−1^ from 0 to −1.6 V_RHE_. All recorded electrode potentials versus Ag/AgCl were converted to the RHE reference scale. The electrocatalytic reduction of nitrate was performed in an H‐type electrolytic cell divided by a Nafion 117 membrane. The chronoamperometric method was employed to apply a constant potential for 60 min and the corresponding current density of the self‐supporting Ru‐LIG electrode was recorded, with Ag/AgCl reference electrode and Pt sheet counter electrode. The anodic chamber electrolyte contained 40 mL of 0.5 M K_2_SO_4_ and the cathode chamber electrolyte for nitrate reduction was 40 mL of 0.5 M K_2_SO_4_ + 100 mM KNO_3_, stirred continuously at a stirring rate of 500 rpm. Both chambers were degassed with Ar gas and maintained in an Ar gas atmosphere. After each chronoamperometric test, 800 µL of the electrolyte solution was extracted from the cathode chamber for subsequent UV–Vis measurement of NO_3_RR products. Dilution of the electrolyte sample prior to analysis was necessary when ammonium or nitrite concentration in the electrolyte was high.

The Faradic efficiency was the percentage of the electric charge consumed for the desired product (ammonium or nitrite) generation in the total applied electricity according to the following formula.

(1) $$FE_{\mathrm{product}}=\left(n_{e}\times n_{\mathrm{product}}\times F\right)/Q\times 100\%$$

where *n*_e_ is the number of electrons required to form the desired product per molecule,^[^ ^49^ ^]^ *n*_product_ is the number of moles of the desired product, F is the Faraday constant (96 485 C mol^−1^), Q is the total amount of charge passed through the working electrode during NO_3_RR.

The yield rate (µg cm^−2^ h^−1^) of ammonium or nitrite was calculated using the following equation.

(2) $$\mathrm{Yield}\mathrm{rate}=m_{\mathrm{product}}/\;\;\left(t\times A\right)$$

where *m*_product_ (µg) is the mass of the desired product, *t* (h) is the reaction time, and A (cm^−2^) is the geometry area of the electrode.

### Determination of Ammonium

The amounts of ammonium in the electrolyte solution were quantitatively determined using the indophenol blue colorimetric method, which includes three reagents prepared in ultrapure water: a) coloring solution of 0.55 M NaOH + 5.0 wt.% sodium citrate + 5.0 wt.% salicylic acid, b) catalyst solution of 1.0 wt.% sodium nitroferricyanide dihydrate, and c) oxidation solution of 0.05 M NaClO. 800 µL of the electrolyte sample was taken from the cathode chamber and diluted to the detection range using ultrapure water. Then, 800 µL of a) coloring solution, 80 µL of b) catalyst solution, and 40 µL of c) oxidizing solution, were added sequentially to the above solution. After a 2 h reaction in the dark, the absorption spectra were recorded with the wavelength ranging from 800 to 500 nm, and the concentrations of ammonium were determined using the absorption peak at ≈665 nm. To plot a calibration curve, the above procedure was applied to a series of standard NH_4_Cl solutions.

### Determination of Nitrite

The produced NO_2_^−^ in the electrolyte was quantitatively detected using the Griess reagent, which was prepared by dissolving 0.1 g of N‐(1‐naphthyl) ethylenediamine dihydrochloride and 1.0 g of sulfonamide in 50 mL ultrapure water containing 2.94 mL H_3_PO_4_. Next, 400 µL Griess reagent and 400 µL diluted electrolyte were added to 800 µL ultrapure water and allowed to react in the dark for 15 min. The above‐mixed solution was then subjected to UV–Vis measurements and the absorption peak at ca. 540 nm was used to detect the concentration of NO_2_^−^. The concentration‐absorbance curve was obtained by plotting the absorbance of a series of standard NaNO_2_ solutions.

## Conflict of Interest

The authors declare no conflict of interest.

## Author Contributions

Z.G. performed methodology, investigation, validation, and visualization, and wrote the original draft. Z.F. performed methodology, and investigation, Wrote, reviewed, and edited the original draft. H.K., J.S., K.Z., and J.L. performed methodology, investigation, and validation. X.L. performed supervision, and visualization and wrote, reviewed, and edited the original draft. L.G. performed conceptualization, methodology, and supervision, Wrote, reviewed, and edited the original draft. P.G. performed investigation, validation, and funding acquisition and wrote, reviewed, and edited the original draft. F.L. performed supervision, funding acquisition, and project administration and wrote, reviewed, and edited the original draft.

## Supporting information

Supporting Information

## Data Availability

The data that support the findings of this study are available from the corresponding author upon reasonable request.
