# Supplementary material for: Ruthenium Anchored Laser‐Induced Graphene as Binder‐Free and Free‐Standing Electrode for Selective Electrosynthesis of Ammonia from Nitrate
Source: Adv Sci (Weinh). 2024 Aug 13;11(39):2406843. doi: 10.1002/advs.202406843 (PMC11497038; doi:10.1002/advs.202406843)
Supplement: Supplementary file 1 — Supporting Information [file ADVS-11-2406843-s001.pdf]

ADVANCED SCIENCE
Open Access

Supporting Information

for *Adv. Sci.*, DOI 10.1002/advs.202406843

Ruthenium Anchored Laser-Induced Graphene as Binder-Free and Free-Standing Electrode for Selective Electrosynthesis of Ammonia from Nitrate

Zekun Geng, Zhiliang Feng, Haoran Kong, Jiaqi Su, Kaiyan Zhang, Jiaxin Li, Xinzhi Sun, Xiaojuan Liu, Lei Ge, Panpan Gai* and Feng Li**

Supporting Information

Ruthenium Anchored Laser-Induced Graphene as Binder-Free and Free-Standing Electrode for Selective Electrosynthesis of Ammonia from Nitrate

Zekun Geng,[a] Zhiliang Feng,[a] Haoran Kong,[a] Jiaqi Su,[a] Kaiyan Zhang,[a] Jiaxin Li,[a] Xinzhi Sun,[a] Xiaojuan Liu,[a] Lei Ge,[a,b,]* Panpan Gai,[a,]* and Feng Li[a]*

[a]College of Chemistry and Pharmaceutical Sciences, Qingdao Agricultural University, Qingdao, 266109, China

[b]Key Laboratory of Advanced Energy Materials Chemistry (Ministry of Education), Nankai University, Tianjin 300071, China

*Corresponding author: Feng Li, Panpan Gai, Lei Ge

E-mail: lifeng@qau.edu.cn; ppgai@qau.edu.cn; lge@qau.edu.cn

Telephone: +86-532-86080855

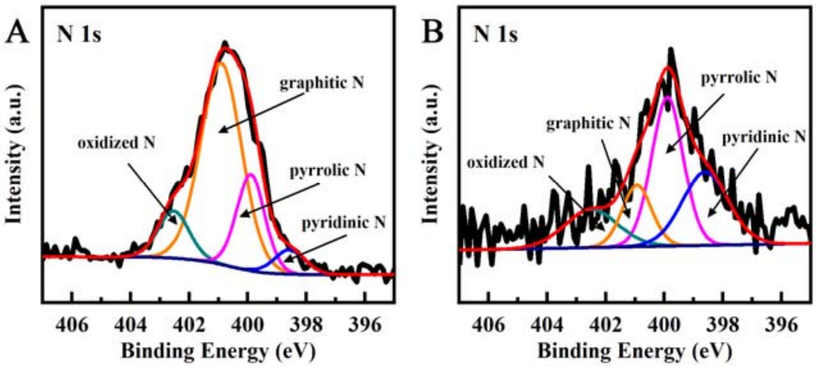

Figure S1. High-resolution N 1s XPS of (A) LIG@(+2,12%) and (B) Ru-LIG@(+2,12%) electrode.

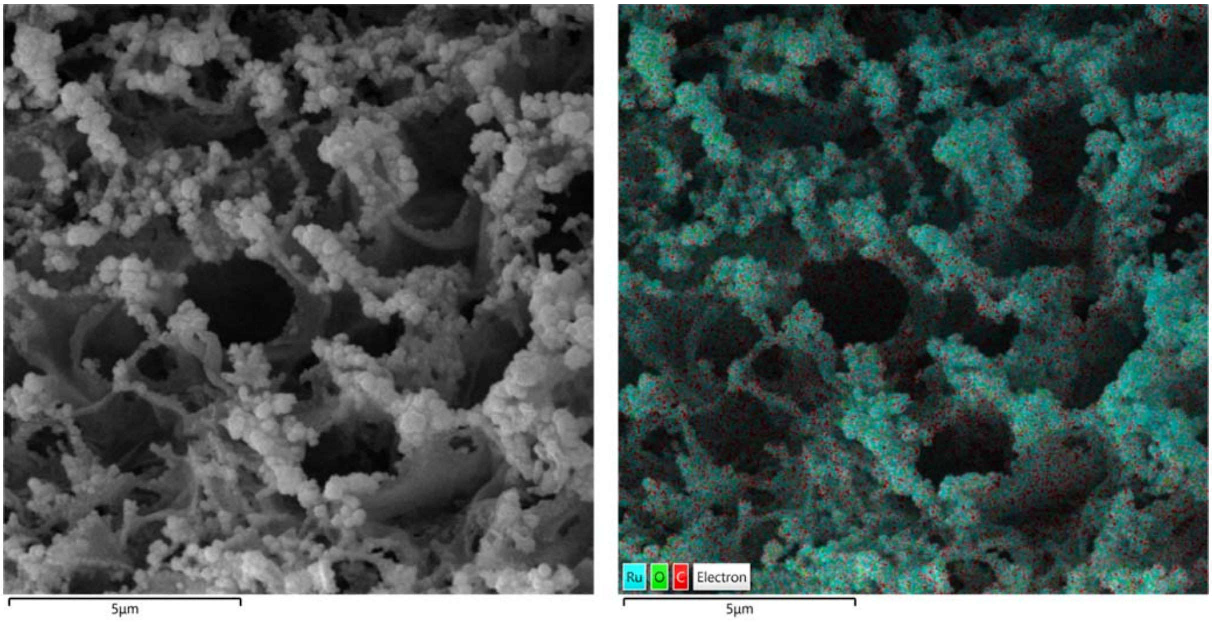

Figure S2. Elemental mapping image of Ru-LIG@(+2,12%) electrode.

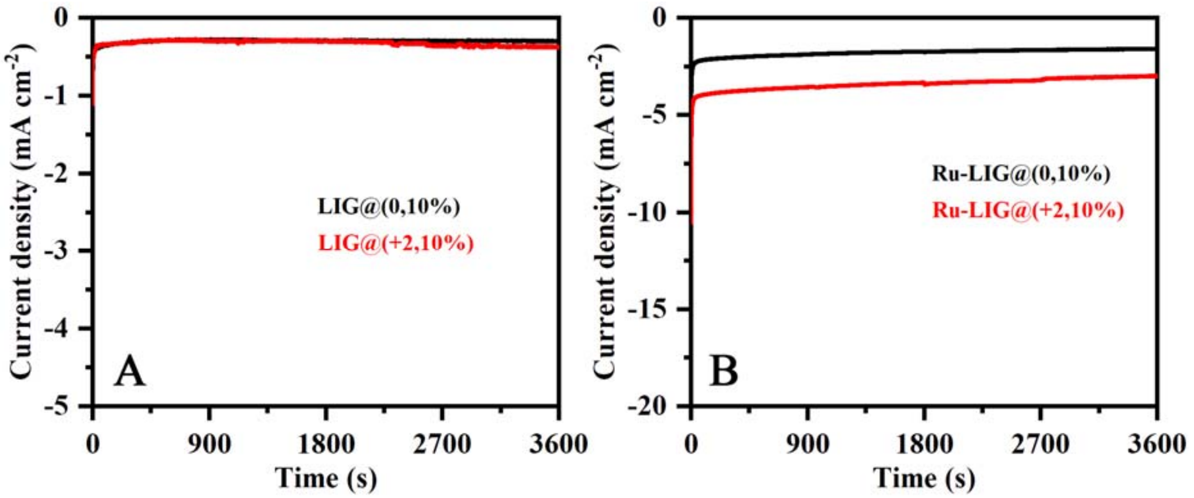

Figure S3. Chronoamperometric curves of (A) LIG@(x,10%) electrodes and (B) Ru-LIG@(x,10%) electrodes recorded at −0.39 V_{RHE} in Ar-saturated 0.5 M K_2SO_4 electrolyte with 100 mM KNO_3.

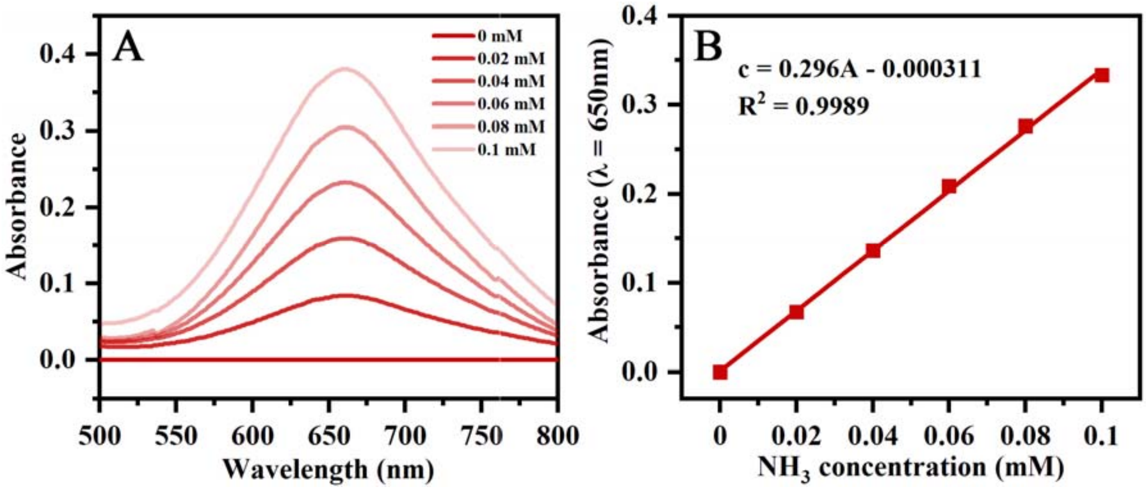

Figure S4. (A) UV-Vis absorption spectra and (B) standard curves of NH_3 for different concentrations.

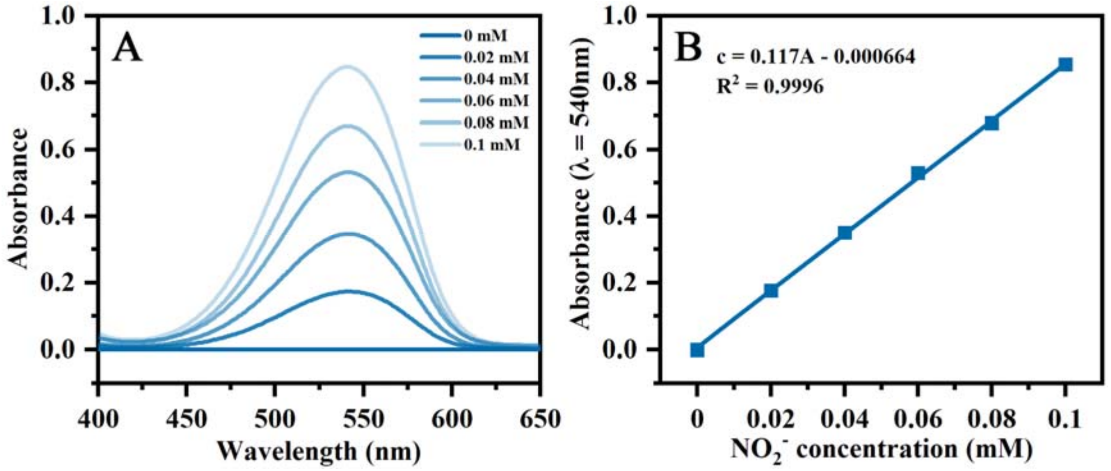

Figure S5. (A) UV-Vis absorption spectra and (B) standard curves of NO_2^- for different concentrations.

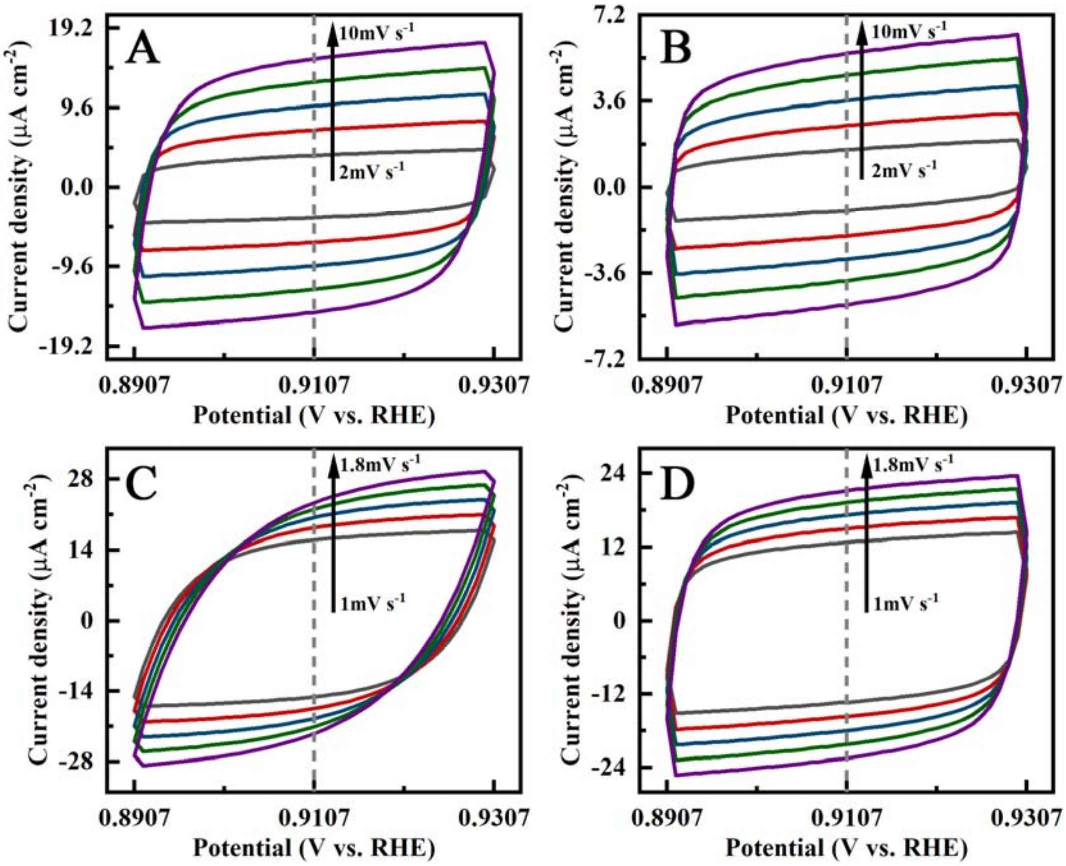

Figure S6. CV profiles of (A) LIG@(0,10%), (B) LIG@(+2,10%), (C) Ru-LIG@(0,10%), and (D) Ru-LIG@(+2,10%) electrodes measured in a non-Faradaic region of the voltammogram at the following scan rate: 2, 4, 6, 8, and 10 mV/s for LIG electrodes and 1.0, 1.2, 1.4, 1.6, and 1.8 mV/s for Ru-LIG electrodes in Ar-saturated 0.5 M K_2SO_4 electrolyte.

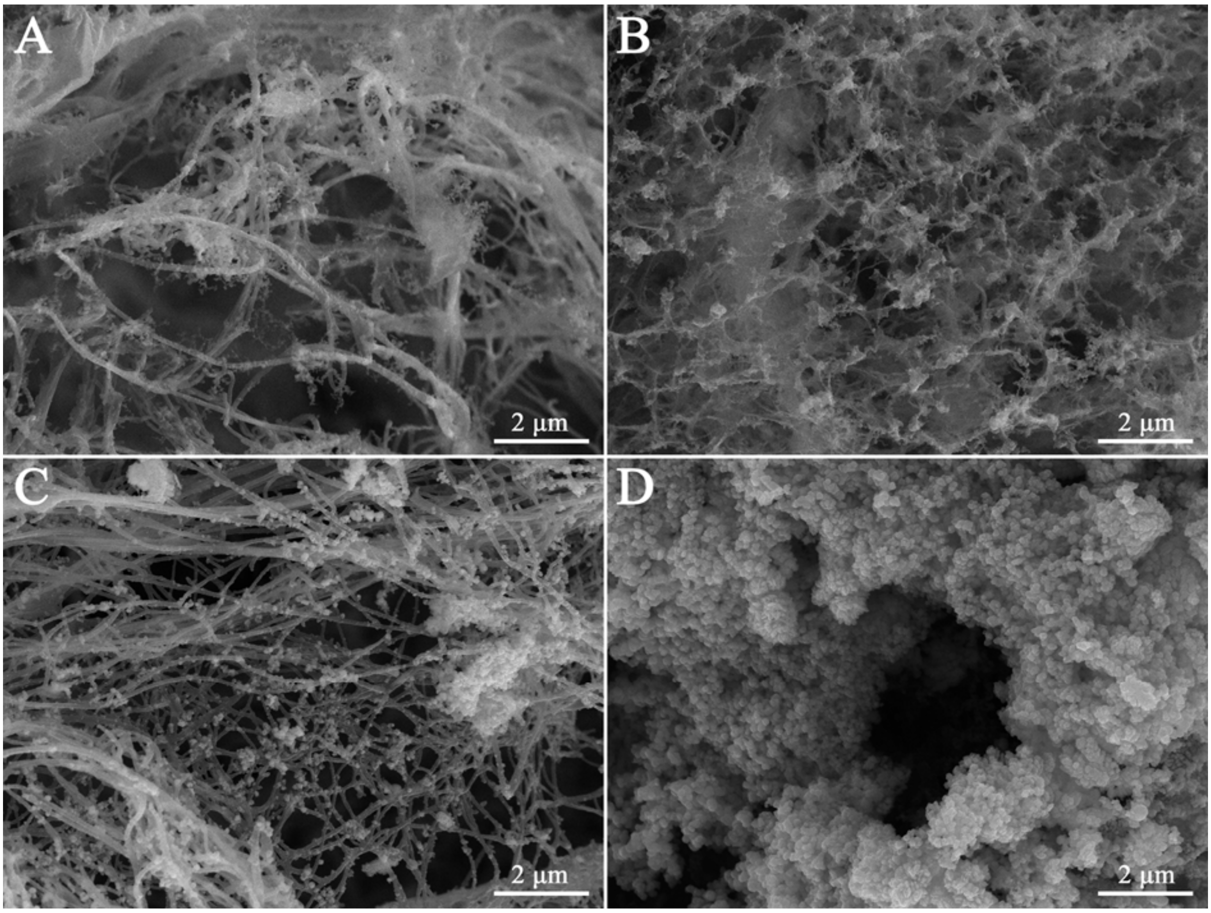

Figure S7. SEM images of (A) LIG@(0,10%), (B) LIG@(+2,10%), (C) Ru-LIG@(0,10%), and (D) Ru-LIG@(+2,10%) electrodes at high magnification.

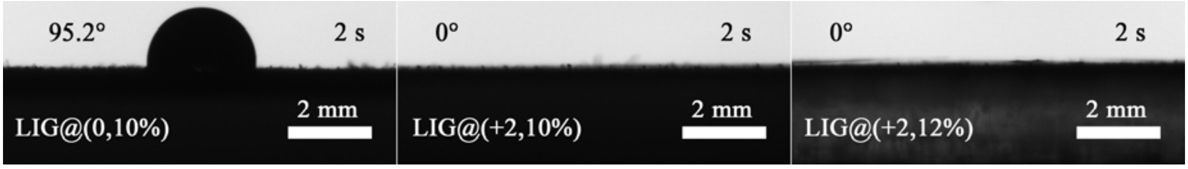

Figure S8. Hydrophilicity of LIG@(0,10%), LIG@(+2,10%), and LIG@(+2,12%).

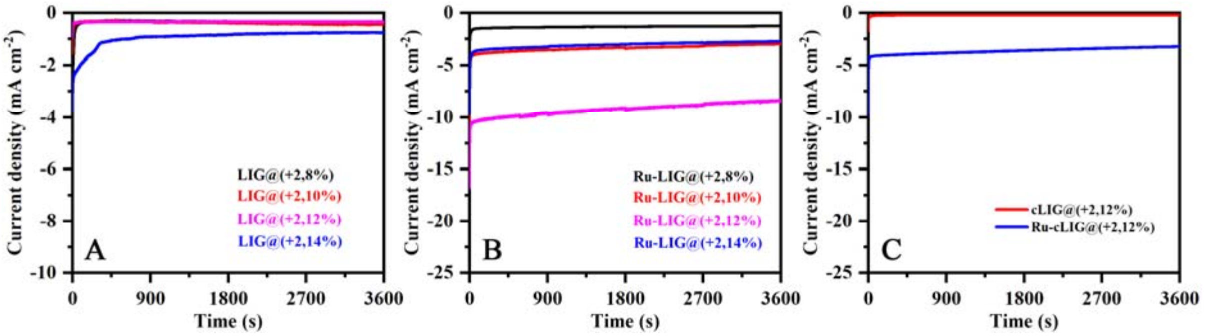

Figure S9. Chronoamperometric curves of (A) LIG@(+2,p) electrodes and (B) Ru-LIG@(+2,p) electrodes recorded at −0.39 V_{RHE} in Ar-saturated 0.5 M K_2SO_4 electrolyte with 100 mM KNO_3. (C) Chronoamperometric curves of cLIG@(+2,12%) electrodes and Ru-cLIG@(+2,12%) electrodes recorded at −0.39 V_{RHE} in Ar-saturated 0.5 M K_2SO_4 electrolyte with 100 mM KNO_3.

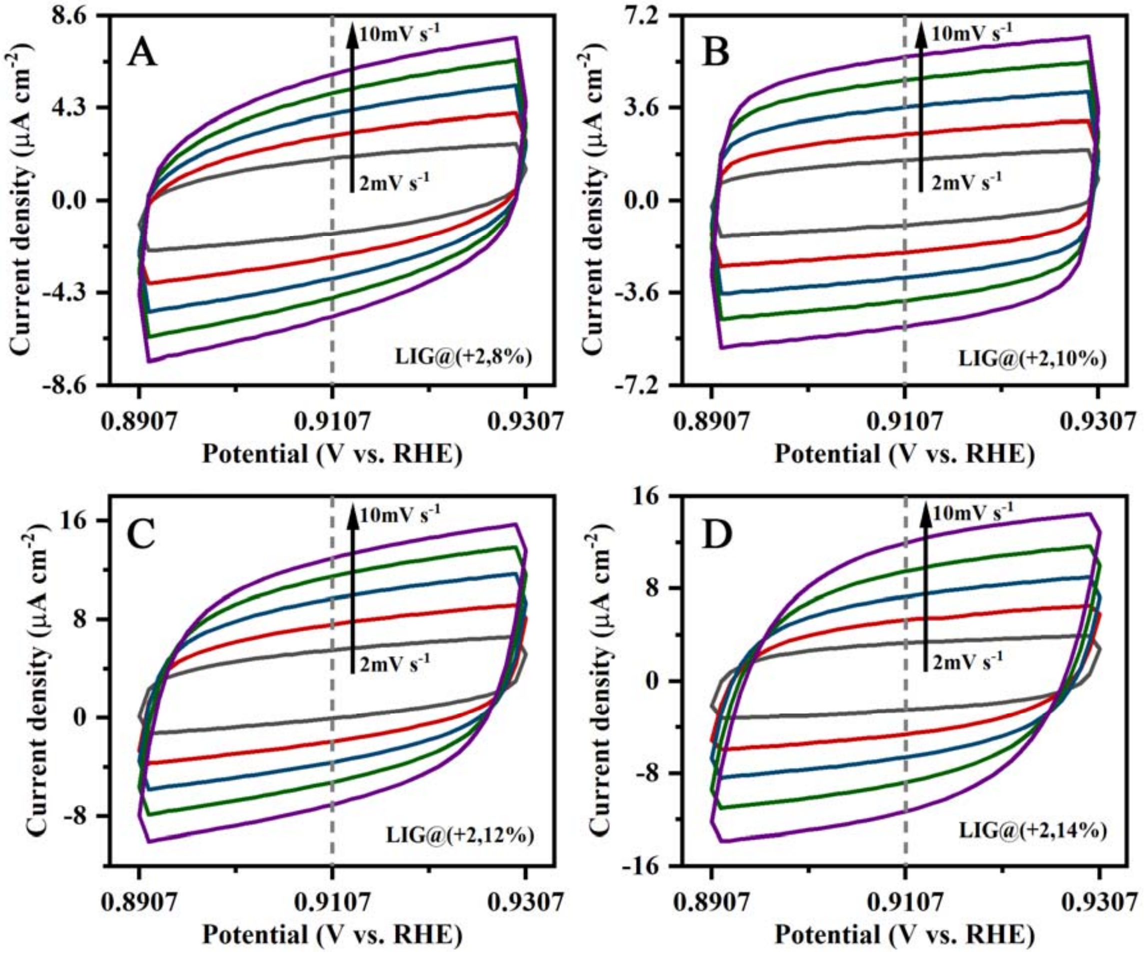

Figure S10. CV profiles of (A) LIG@(+2,8%), (B) LIG@(+2,10%), (C) LIG@(+2,12%), and (D) LIG@(+2,14%) electrodes measured in a non-Faradaic region of the voltammogram at the following scan rate: 2, 4, 6, 8, and 10 mV/s in Ar-saturated 0.5 M K_2SO_4 electrolyte.

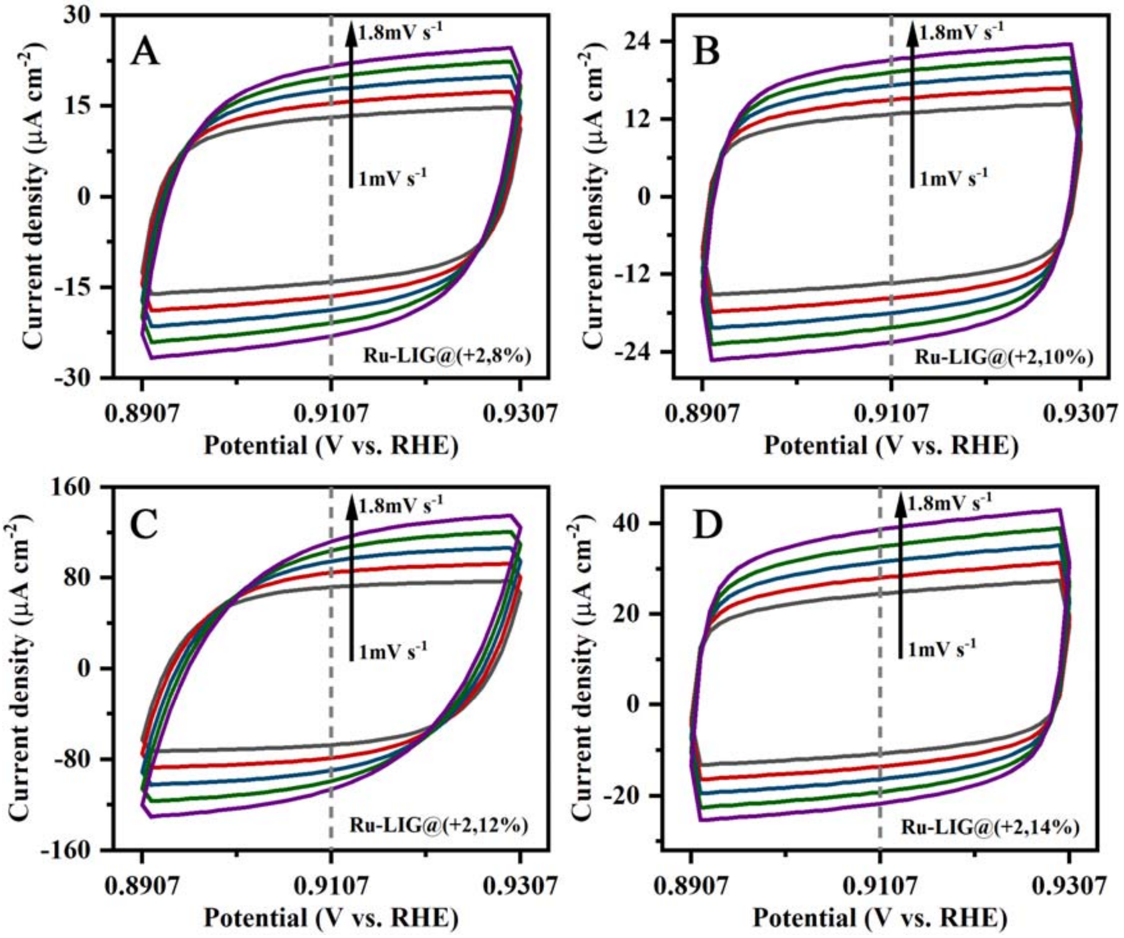

Figure S11. CV profiles of (A) Ru-LIG@(+2,8%), (B) Ru-LIG@(+2,10%), (C) Ru-LIG@(+2,12%), and (D) Ru-LIG@(+2,14%) electrodes measured in a non-Faradaic region of the voltammogram at the following scan rate: 1.0, 1.2, 1.4, 1.6, and 1.8 mV/s in Ar-saturated 0.5 M K_2SO_4 electrolyte.

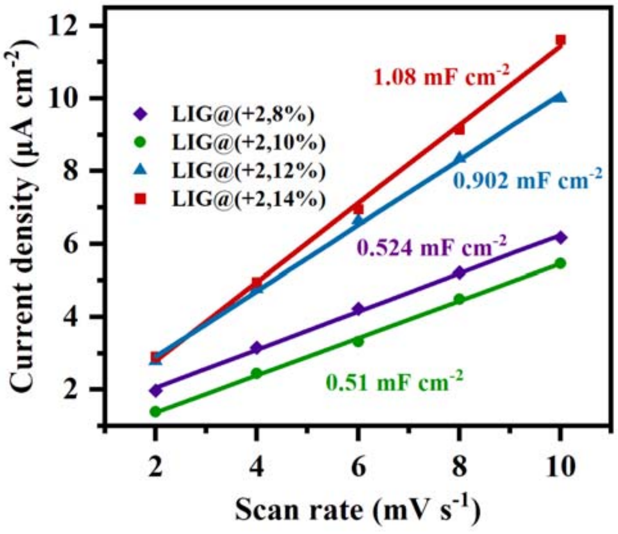

Figure S12. The capacitive currents of LIG@(+2,p) electrodes measured at about 0.91 V_{RHE} plotted as a function of scan rate.

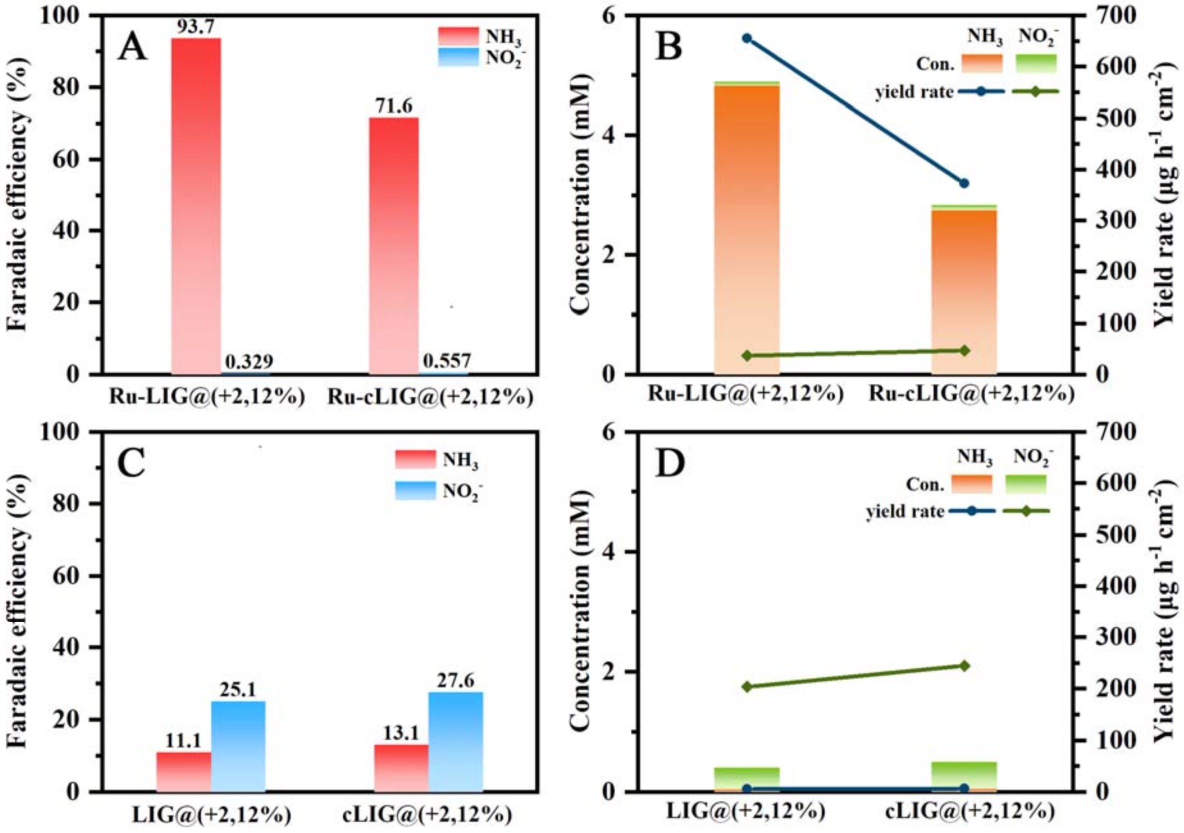

Figure S13. (A,C) Product FEs, (B,D) product distribution and corresponding yield rate of (A,B) Ru-cLIG@(+2,12%) and (C,D) cLIG@(+2,12%) electrodes at an applied potential of −0.59 V_{RHE} in Ar-saturated 0.5 M K_2SO_4 electrolyte with/without 100 mM KNO_3.

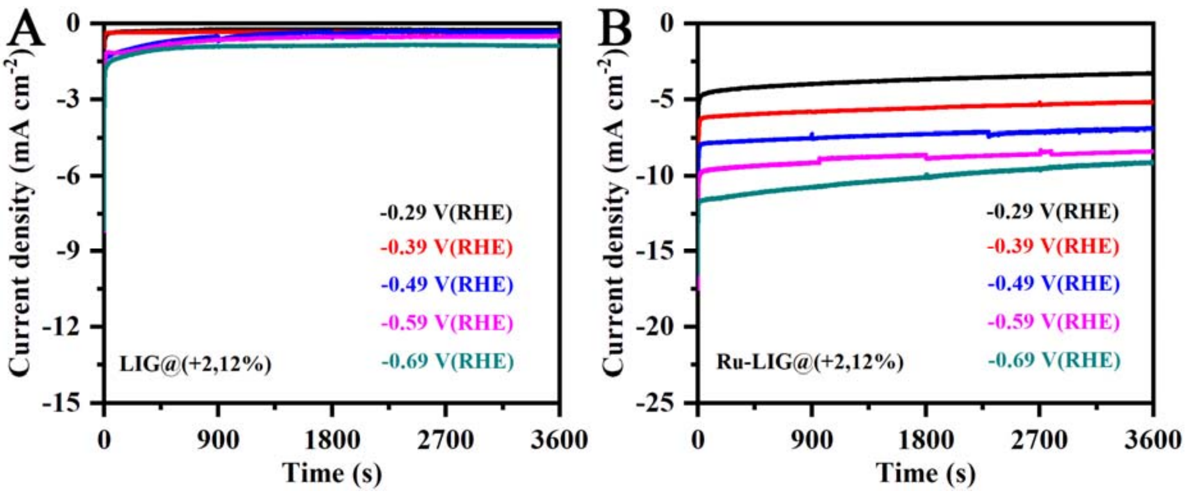

Figure S14. Chronoamperometric curves of (A) LIG@(+2,12%) electrodes and (B) Ru-LIG@(+2,12%) electrodes recorded at different applied potentials in Ar-saturated 0.5 M K_2SO_4 electrolyte with 100 mM KNO_3.

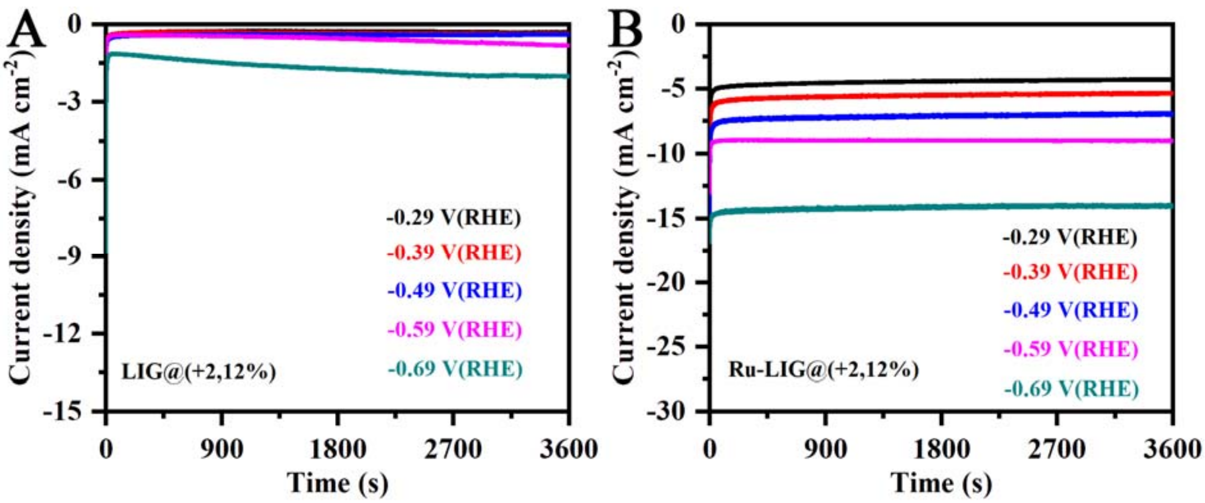

Figure S15. Chronoamperometric curves of (A) LIG@(+2,12%) electrodes and (B) Ru-LIG@(+2,12%) electrodes recorded at different applied potentials in Ar-saturated 0.5 M K_2SO_4 electrolyte with 100 mM $NaNO_2$.

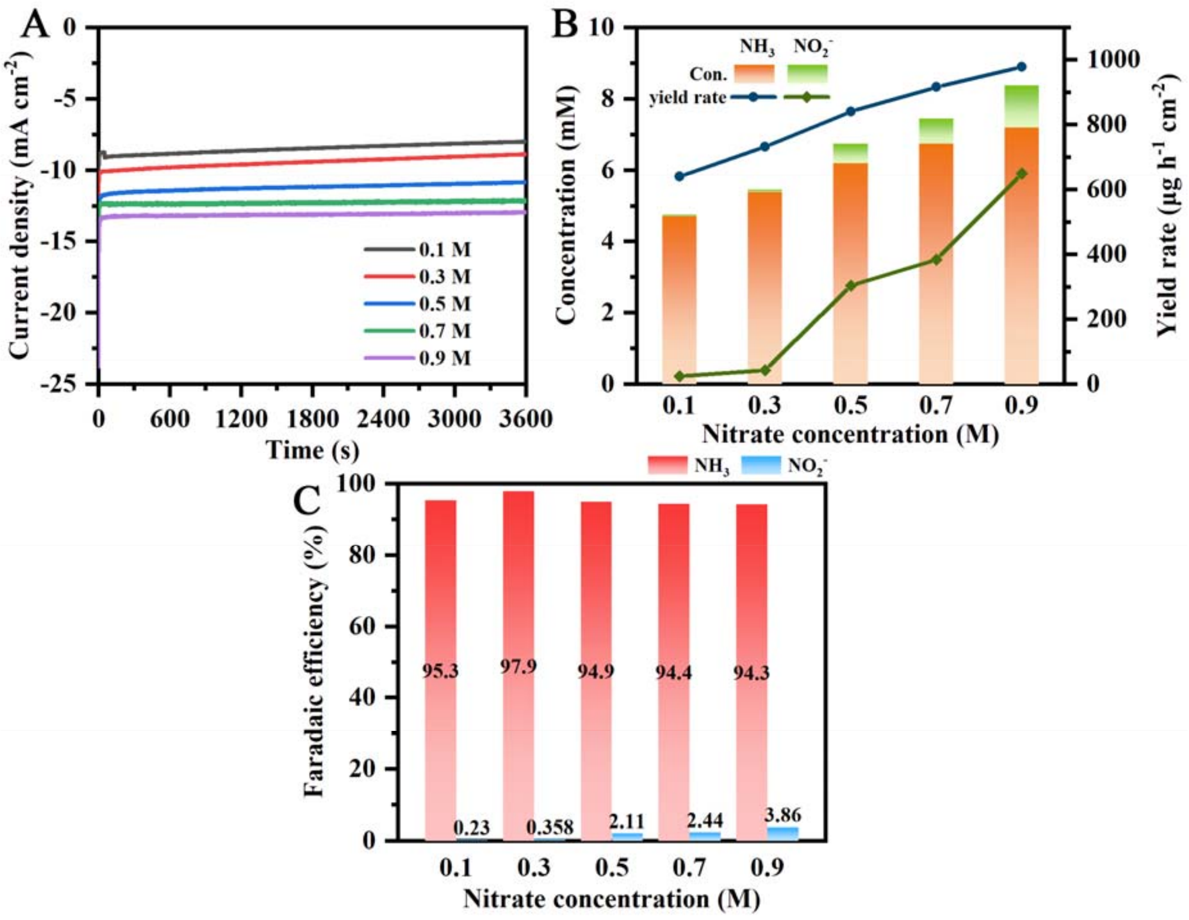

Figure S16. (A) Chronoamperometric curves, (B) product distribution and corresponding yield rate, (C) product FEs of Ru-LIG@(+2,12%) electrode recorded at −0.59 V_{RHE} in Ar-saturated 0.5 M K_2SO_4 electrolyte with different KNO_3 concentrations.

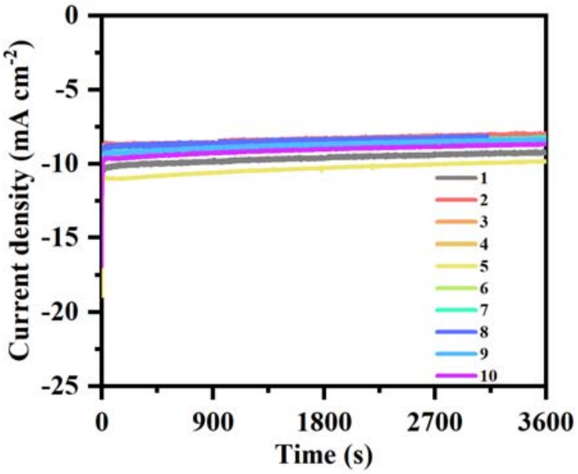

Figure S17. Chronoamperometric curves of Ru-LIG@(+2,12%) electrode recorded for ten consecutive NO_3RR recycling tests at −0.59 V_{RHE} in Ar-saturated 0.5 M K_2SO_4 electrolyte with 100 mM KNO_3.

Table S1. Comparison of the NO_3RR activities of different electrocatalysts under similar conditions.

Electrocatalyst	Electrolyte	pH	Potential (V_{RHE})	NH_3 yield rate (μg cm^{-2} h^{-1})	FE (NH_3)	Ref.
Ru-LIG on PI film	0.5 M K_2SO_4 + 0.1 M KNO_3	7	−0.59	655.9	93.7	**This work**
LIG on Au-coated paper	1.0 M $NaNO_3$	7	-0.63	375	74	[1]
LIG on Au-coated paper	1.0 M $NaNO_3$	7	-0.63	250	60	[2]
CoPB-C8[a] on glassy carbon	0.5 M Na_2SO_4 + 0.1 M $NaNO_3$	7	-0.6	250	95	[3]
$CoMn_2O_4$ on carbon cloth	0.1 M Na_2SO_4 + 0.1 M $NaNO_3$	7	-0.6	410	80	[4]
nickel porphyrin COF on carbon paper	0.5 M K_2SO_4 + 0.1 M KNO_3	7	-0.8	500	80	[5]
Fe SAC on glassy carbon	0.1 M K_2SO_4 + 0.5 M KNO_3	7	-0.59	800	65	[6]
$LaCoO_3$ on carbon paper	1 M Na_2SO_4 + 0.5 M KNO_3	7	-0.8	30.6	85	[7]

red muds on carbon cloth	1 M PBS + 0.1 M KNO_3	7	-0.73	850	69	[8]
Fe_2TiO_5 on carbon paper	0.1 M PBS + 0.1 M $NaNO_3$	7	-0.8	765	85	[9]
Fe-Co_3O_4 on Ti mesh	0.1 M PBS + 0.05 M KNO_3	7	-0.6	500	94	[10]
FeMo—N—C on carbon paper	0.05 M PBS + 0.16 M KNO_3	7	-0.55	391	82	[11]
Cu–PTCDA[b] on carbon cloth	0.1 M PBS + 500 ppm KNO_3	7	-0.6	900	65	[12]
Pd-NDs/Zr-MOF on carbon paper	0.1 M Na_2SO_4 + 500 ppm KNO_3	7	-0.8	88.5	15	[13]
Cu/Cu_2O on carbon paper	0.1 M PBS + 0.1 M KNO_3	7	−0.6	850	50	[14]
Pd/TiO_2 array on carbon cloth	1.0 M LiCl + 0.25 M $LiNO_3$	7	-0.6	500	50	[15]
MnOx on carbon framework	0.5 M Na_2SO_4 + 0.1 M KNO_3	7	-0.61	30	25	[16]

Cu_3P array on carbon cloth	0.1 M PBS + 0.1 M $NaNO_3$	7	-0.6	600	63	[17]
RuNi-MOFs on nickel foam	0.1 M Na_2SO_4 + 50 ppm KNO_3	7	-0.59	170	75	[18]
Ru-N-C on carbon paper	0.05 M PBS + 0.16 M KNO_3	7	-0.6	9	23	[19]

[a]a cobalt porphyrin-based three-dimensional porous organic cage structure (CoPB-C8).

[b]an organic molecular solid (3,4,9,10-perylenetetracarboxylic dianhydride (PTCDA)).

References

[1] L. Huang, L. Cheng, T. Ma, J.-J. Zhang, H. Wu, J. Su, Y. Song, H. Zhu, Q. Liu, M. Zhu, Z. Zeng, Q. He, M.-K. Tse, D.-t. Yang, B. I. Yakobson, B. Z. Tang, Y. Ren, R. Ye, *Adv. Mater.* **2023**, *35*, 2211856.

[2] L. Cheng, T. Ma, B. Zhang, L. Huang, W. Guo, F. Hu, H. Zhu, Z. Wang, T. Zheng, D.-T. Yang, C.-K. Siu, Q. Liu, Y. Ren, C. Xia, B. Z. Tang, R. Ye, *ACS Catal.* **2022**, *12*, 11639.

[3] L. An, M. R. Narouz, P. T. Smith, P. De La Torre, C. J. Chang, *Angew. Chem. Int. Ed.* **2023**, *62*, e202305719.

[4] Z. Niu, S. Fan, X. Li, J. Duan, A. Chen, *Appl. Catal. B* **2023**, *322*, 122090.

[5] F. Lv, M. Sun, Y. Hu, J. Xu, W. Huang, N. Han, B. Huang, Y. Li, *Energy Environ. Sci.* **2023**, *16*, 201.

[6] Z.-Y. Wu, M. Karamad, X. Yong, Q. Huang, D. A. Cullen, P. Zhu, C. Xia, Q. Xiao, M. Shakouri, F.-Y. Chen, J. Y. Kim, Y. Xia, K. Heck, Y. Hu, M. S. Wong, Q. Li, I. Gates, S. Siahrostami, H. Wang, *Nat. Commun.* **2021**, *12*, 2870.

[7] H. Zheng, Y. Zhang, Y. Wang, Z. Wu, F. Lai, G. Chao, N. Zhang, L. Zhang, T. Liu, *Small* **2023**, *19*, 2205625.

[8] Y.-T. Xu, K.-C. Ren, Z.-M. Tao, D. K. Sam, E. Feng, X. Wang, G. Zhang, J. Wu, Y. Cao, *Green Chem.* **2023**, *25*, 589.

[9] H. Du, H. Guo, K. Wang, X. Du, B. A. Beshiwork, S. Sun, Y. Luo, Q. Liu, T. Li, X. Sun, *Angew. Chem. Int. Ed.* **2023**, *62*, e202215782.

[10] P. Wei, J. Liang, Q. Liu, L. Xie, X. Tong, Y. Ren, T. Li, Y. Luo, N. Li, B. Tang, A. M. Asiri, M. S. Hamdy, Q. Kong, Z. Wang, X. Sun, *J. Colloid Interface Sci.* **2022**, *615*, 636.

[11] E. Murphy, Y. Liu, I. Matanovic, S. Guo, P. Tieu, Y. Huang, A. Ly, S. Das, I. Zenyuk, X. Pan, E. Spoerke, P. Atanassov, *ACS Catal.* **2022**, *12*, 6651.

[12] G.-F. Chen, Y. Yuan, H. Jiang, S.-Y. Ren, L.-X. Ding, L. Ma, T. Wu, J. Lu, H. Wang, *Nat. Energy* **2020**, *5*, 605.

[13] M. Jiang, J. Su, X. Song, P. Zhang, M. Zhu, L. Qin, Z. Tie, J.-L. Zuo, Z. Jin, *Nano Lett.* **2022**, *22*, 2529.

[14] N. Zhou, Z. Wang, N. Zhang, D. Bao, H. Zhong, X. Zhang, *ACS Catal.* **2023**, *13*, 7529.

[15] Y. Guo, R. Zhang, S. Zhang, Y. Zhao, Q. Yang, Z. Huang, B. Dong, C. Zhi, *Energy Environ. Sci.* **2021**, *14*, 3938.

[16] W. Gao, J. V. Perales-Rondon, J. Michalička, M. Pumera, *Appl. Catal. B* **2023**, *330*, 122632.

[17] J. Liang, B. Deng, Q. Liu, G. Wen, Q. Liu, T. Li, Y. Luo, A. A. Alshehri, K. A. Alzahrani, D. Ma, X. Sun, *Green Chem.* **2021**, *23*, 5487.

[18] J. Qin, K. Wu, L. Chen, X. Wang, Q. Zhao, B. Liu, Z. Ye, *J. Mater. Chem. A* **2022**, *10*, 3963.

[19] E. Murphy, Y. Liu, I. Matanovic, M. Rüscher, Y. Huang, A. Ly, S. Guo, W. Zang, X. Yan, A. Martini, J. Timoshenko, B. R. Cuenya, I. V. Zenyuk, X. Pan, E. D. Spoerke, P. Atanassov, *Nat. Commun.* **2023**, *14*, 4554.
